# Efficacy and safety of blinatumomab in children with relapsed/refractory B cell acute lymphoblastic leukemia: A systematic review and meta-analysis

**DOI:** 10.3389/fphar.2022.1032664

**Published:** 2023-01-10

**Authors:** Bin Chen, Zhuan Zou, Qian Zhang, Kexing Chen, Xiaoyan Zhang, Dongqiong Xiao, Xihong Li

**Affiliations:** ^1^ Department of Pediatrics, West China Second University Hospital, Sichuan University, Chengdu, China; ^2^ Key Laboratory of Birth Defects and Related Diseases of Women and Children, Sichuan University, Chengdu, China

**Keywords:** blinatumomab, relapsed/refractory acute lymphoblastic leukemia (R/R ALL), child, meta-analysis, systemic review

## Abstract

**Objectives:** Several clinical trials have been conducted to evaluate the effects of blinatumomab in childhood B cell acute lymphoblastic leukemia (B-ALL). We conducted this meta-analysis to validate the efficacy and safety of blinatumomab in pediatric patients with relapsed/refractory B-ALL (R/R B-ALL).

**Methods:** We searched and investigated all relevant studies in the PubMed, Web of Science, Embase, and Cochrane Library databases. The primary outcomes were complete response (CR), overall survival (OS), event free survival (EFS), minimal residual disease (MRD) response, allogeneic hematopoietic stem cell transplantation (allo-HSCT) and were calculated separately for randomized controlled trials (RCTs) and single-arm studies. The secondary end points were adverse effects (AEs) and the relapse rate. The Cochrane, bias assessment tool, was used to assess the risk of bias in RCTs. The methodological quality of single-arm studies was assessed using the methodological index for non-randomized studies (MINORS) tool.

**Results:** The meta-analysis included two RCTs and 10 single-arm studies, including 652 patients in total. Our study showed that in the single-arm studies, the combined CR rate was 0.56 (95% confidence interval (CI): 0.45 -0.68), the odds ratios (ORs) of OS was 0.43 (95% CI 0.32 -0.54), the EFS rate was 0.30 (95% CI: 0.20 -0.40), the MRD response was 0.51 (95% CI: 0.34 -0.68), allo-HSCT rate was 0.62 (95% CI: 0.50 -.74), the AE rate was 0.65 (95% CI: 0.54 -0.76) and the relapse rate was 0.32 (95% CI: 0.27 -0.38). In the RCTs, the blinatumomab-treated group compared with the chemotherapy group had a combined OS rate of 0.12 (95% CI: 0.05 -0.19) and an EFS rate of 2.16 (95% CI: 1.54 -3.03). The pooled MRD response rate was 4.71 (95% CI:2.84 -7.81), allo-HSCT was 3.24 (95% CI: 1.96 -5.35), the AE rate was 0.31 (95% CI: 0.16 -0.60), and the relapse rate was 0 .69 (95% CI: 0.43 -1.09).

**Conclusion:** According to this meta-analysis, blinatumomab shows potent therapeutic efficacy and limited AEs in children with R/R B- ALL.

**Systematic Review Registration:** https://www.crd.york.ac.uk/prospero/, identifier CRD42022361914.

## 1 Introduction

B cell Acute lymphoblastic leukemia (B-ALL) is a common and life-threatening hematological malignancy. Relapse is the main cause of treatment failure in children with ALL (Bhojwani and Pui, 2013). Long-term survival rates after relapse remain below 50% (Locatelli et al., 2012). Relapsed/refractory ALL (R/R ALL) has an extremely poor prognosis, The prognosis of relapse after allogeneic hematopoietic stem cell transplantation (allo-HSCT), second or subsequent relapse, or failure of second-line salvage chemotherapy is dismal, with 2- to 3-year survival rates of ≤20% (Kuhlen et al., 2018; Stein et al., 2019), 13%–27% (Ko et al., 2010), and <10% (von Stackelberg et al., 2011), respectively. The standard regimen for first relapse treatment consists of 4 weeks of reinduction chemotherapy, followed by consolidation therapy, which includes two cycles of intensive multiagent chemotherapy for early bone marrow (BM) relapse (<36 months after diagnosis), followed by hematopoietic stem cell transplantation (HSCT) (Locatelli et al., 2012). Patients with high-risk first-relapse ALL are candidates to receive allo-HSCT when a second cytomorphologic complete remission is achieved; allo-HSCT is a very effective approach for preventing further recurrence in these patients (Peters et al., 2015).

Blinatumomab is a bispecific T cell–engaging antibody construct that links CD3^+^ T cell to CD19^+^ leukemia cells and engages T cell to lyse CD19-expressing B cell (Bargou et al., 2008), inducing a cytotoxic immune response. In 2014, blinatumomab was approved by the United States Food and Drug Administration for the treatment of adults and children with Relapsed/refractory B-ALL (R/R B-ALL) (Kantarjian et al., 2017). The efficacy and safety of blinatumomab in R/R B-ALL have varied widely across clinical trials (Topp et al., 2015; Goebeler et al., 2016; Viardot et al., 2016; Kantarjian et al., 2017; Martinelli et al., 2017). Blinatumomab, is active in relapsed and refractory (R/R) adult and pediatric ALL (Hunger and Raetz, 2020). Children’s Oncology Group (COG) AALL1331, which compared two cycles of United Kingdom (UKALL) R3 postinduction chemotherapy to two cycles of blinatumomab, was recently stopped early due to improved disease-free survival (DFS), superior overall survival (OS), lower toxicity and superior minimal residual disease (MRD) clearance (Brown et al., 2019). Yu et al. (2019) performed a meta-analysis to confirm that blinatumomab effectively treats R/R B-ALL in adults. Marrapodi et al. (2022) performed a meta-analysis to investigate the safety of blinatumomab in the treatment of childhood ALL. However, there are currently no relevant comprehensive meta-analyses of the efficacy and safety of blinatumomab in the treatment of children with R/R B-ALL.

We conducted this meta-analysis to provide more comprehensive evidence on the efficacy and safety of blinatumomab in children with R/R B cell ALL.

## 2 Methods

The literature review was carried out according to the reporting items for systematic reviews and meta-analyses (PRISMA) guidelines (Moher et al., 2009).

### 2.1 Search strategy and information extraction

We searched all articles in PubMed, Web of Science, Embase, and Cochrane Library until 16 July 2022, using “blinatumomab,” “blincyto,” or “MT103” as search terms in the title and abstract. All completed clinical trials and single-arm studies of blinatumomab in children with R/R B-ALL were included. Supplementary Figure S1 displays the search strategy applied in this meta-analysis. The retrieved studies were imported into the EndNoteX9 software (Clarivate Analytics, London, United Kingdom), and the full texts of the articles that met the inclusion/exclusion criteria were downloaded and read. We developed an information extraction spreadsheet for this project, including title, first author, year of publication, study type, author country, patient characteristics (age, sex, number of patients), dose in the experimental group, follow-up period, and outcome indicators.

Two researchers (CB and CKX) independently conducted the appeal literature screening and information extraction. After completion, the two researchers cross-examined each other. A third researcher (XDQ) will assist in adjudication if there is a dispute.

### 2.2 Inclusion and exclusion criteria

The inclusion criteria were: 1) participants: children and adolescents with R/R B cell ALL, 2) intervention: treatment with blinatumomab, and 3) outcomes: data on survival outcomes, responses, and treatment-related adverse events were available. The exclusion criteria were: 1) a review or meta-analysis or a case report or conference abstracts, 2) *in-vitro* and animal experiments and 3) the study subjects were adults (Adults are defined as those who are older than 18).

### 2.3 Quality assessment

The quality of the included RCTs was assessed using the Cochrane Collaboration risk of bias assessment tool, which evaluates the risk of bias based on seven items in the following six domains: 1) Selection bias (random sequence generation, allocation concealment), 2) performance bias (Blinding of participants and personnel), 3) detection bias (Blinding of outcome assessment), 4) attrition bias (Incomplete outcome data), 5) reporting bias (selective reporting), 6) other bias. The researchers evaluated the RCT studies item by item, and the evaluation results were expressed as low risk, high risk, or unclear (Higgins et al., 2011). The methodological quality of single-arm studies was assessed with using methodological index for non-randomized studies (MINORS) tool. The MINORS tool consists of eight items for non-comparative studies: 1) a clearly stated aim, 2) inclusion of consecutive patients, 3) prospective collection of data 4) endpoints appropriate to the aim of the study, 5) unbiased assessment of the study endpoint, 6) follow-up period appropriate to the aim of the study, 7) loss to follow up less than 5%, 8) prospective calculation of the study size. An item was scored “0” when not reported, “1” when inadequately reported, and “2” when adequately reported (Slim et al., 2003). The maximum score was 16.

### 2.4 Outcome measures

The primary endpoints were CR (defined as <5% blasts in the bone marrow), OS (defined as the time from the first blinatumomab administration and the last follow-up or death for any reason), event-free survival (EFS; defined as time from the first blinatumomab infusion to relapse, progression, second malignant neoplasm, death or last contact), MRD response [defined as<1 × 10^−4^ leukemic cells in the bone marrow (BM) by flow cytometry (FC) or polymerase chain reaction (PCR) analysis], and allo-HSCT, and were calculated separately for RCTs and single-arm studies. The secondary end points were adverse events (AEs) and relapse rates.

### 2.5 Statistical analysis

All statistical analyses were conducted using the STATA software (Stata-Corp LLC, College Station, TX, United States). The included studies used odds ratios (ORs) and corresponding 95% confidence intervals (CIs) for dichotomous variables or outcomes to evaluate the difference and dichotomous variables. Statistical heterogeneity between the studies was assessed using the Q and I^2^ statistics. When I^2^ > 50% and *p* <.1, indicating high heterogeneity, the random-effects model was used. When I^2^ < 50% and *p* >.1, indicating low heterogeneity, the fixed-effects model was used. Egger’s test was used to evaluate publication bias. Subgroup analysis was conducted to analyze the heterogeneity between studies. *p* <.05 was considered statistically significant.

## 3 Results

### 3.1 Literature search and patient characteristics

**FIGURE 1:**
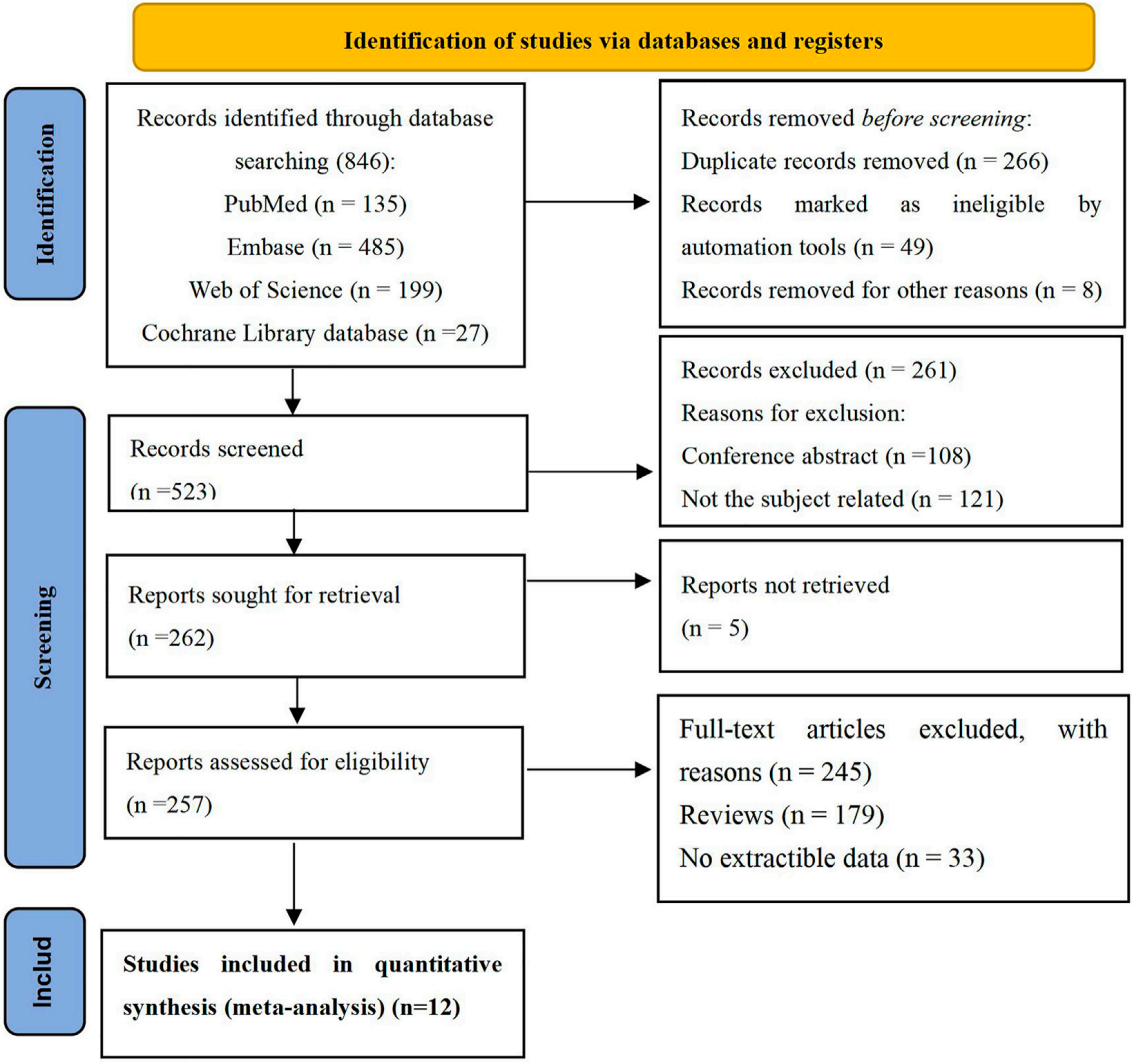
Flow chart of the literature search.

We identified 846 potential studies, including 135 from PubMed, 485 from Embase, 199 from Web of Science, and 27 from the Cochrane Library. After the removal of duplicates, 523 articles were recruited. After reviewing the titles and abstracts, 262 articles were selected for full-text review. Finally, 652 participants from two RCTs and 10 single-arm studies were eligible for inclusion in this meta-analysis (Schlegel et al., 2014; von Stackelberg et al., 2016; Ampatzidou et al., 2020; Fuster et al., 2020; Horibe et al., 2020; Brown et al., 2021; Locatelli et al., 2021; Queudeville et al., 2021; Sutton et al., 2021; Beneduce et al., 2022; Locatelli et al., 2022; Pawinska-Wasikowska et al., 2022). A flow chart of the literature screening is shown in Figure 1. The two RCT studies eligible for inclusion in this meta-analysis were published in JAMA journals in 2021. One RCT study recruited 108 children of aged 1–17 years (Locatelli et al., 2021). The other RCT study enrolled 208 patients, including 175 children aged 1–17 years (Brown et al., 2021). Both studies used a fixed dose of blinatumomab of 15 μg/m^2^/day, and the control group was chemotherapy. The sample size of the single-arm studies ranged from nine to 110, with blinatumomab treatment doses ranging of 5–28 μg/m^2^/day (Schlegel et al., 2014; von Stackelberg et al., 2016; Ampatzidou et al., 2020; Fuster et al., 2020; Horibe et al., 2020; Queudeville et al., 2021; Sutton et al., 2021; Beneduce et al., 2022; Locatelli et al., 2022; Pawinska-Wasikowska et al., 2022). Two studies didn’t report patients age (Fuster et al., 2020; Sutton et al., 2021). One study didn’t report the blinatumomab treatment doses (Fuster et al., 2020). Three studies didn’t report the follow-up time (Ampatzidou et al., 2020; Fuster et al., 2020; Locatelli et al., 2022). All patients enrolled the studies had primary R/R disease after traditional chemotherapy or HSCT. Blinatumomab administered through continuous intravenous infusion. All patients received glucocorticoid prophylaxis before blinatumomab administration. The characteristics of the 12 studies included are summarized in Table 1.

**TABLE 1:** Characteristics of the studies included in the meta-analysis.

RCT									
Study	Country	Sample size (female)		Age (years IQR)		Intervene		Follow up (months IQR)	Out come
		EG	CG	EG	CG	EG (does)	CG		
Locatelli et al. (2021)	Italy	54 (24)	54 (32)	6 (1–17)	5 (1–17)	15 ug/m^2^/day	Chemotherapy	22.4 (8.1–34.2)	M1; M2; M3; M4; M5;
Brown et al. (2021)	United States	105 (48)	103 (49)	6 (3–13)	6 (3–13)	15 ug/m^2^/day	Chemotherapy	34.8 (21.6–46.8)	M1; M2; M3; M4; M5
Single-arm study									
Study	Country	Sample size (female)		Age (years IQR)		Dose		Follow up	Out come
Beneduce et al. (2022)	Italy	39 (17)		5.3 (.2–20.4)		5 to 28 μg/m^2^/day		16 (0–67)	M1; M2; M3; M4; M5; M6;
Wasikowska (2022)	Poland	13 (5)		5.0 (.67–10)		5 to 15 mcg/m^2^/day		25.4 (1–47)	M2; M3; M5; M6
Locatelli et al. (2022)	Italy	110 (48)		8.5 (.4–17.0)		5 to 15 ug/m^2^/day		NR	M1; M2; M3; M6
Horibe et al. (2020)	Japan	9 (5)		11 (7–17)		5 to 15 ug/m^2^/day		24	M2; M3; M5; M6
Αmpatzidou et al. (2020)	Greece	9 (4)		4.1 (.2–12.1)		5 to 45 ug/m^2^/day		NR	M1; M2; M3; M5; M6
Sutton et al. (2020)	Australia	24		NR		15 ug/m^2^/day		26 (14–42)	M1; M2; M3; M5; M6
Queudeville et al. (2020)	Germany	38 (14)		9.8 (1.1–20.7)		5 to 30 ug/m^2^/day		54 (8.9–113)	M1; M2; M3; M4; M5; M6
Schlegel et al. (2014)	Germany	9 (4)		10.4 (4.3–18.5)		5 to 30 ug/m^2^/day		49.7 (22.5–61.7)	M1; M3; M5; M6
Stackelberg (2016)	Germany	70 (23)		8 (<1–17)		5 to 15 ug/m^2^/day		23.8	M1; M2; M3; M5; M6
Fuster et al. (2020)	Spain	15				NR		NR	M1; M2; M3; M5; M6

Abbreviations: RCT, Randomized controlled trial; EG, Blinatumomab group; CG, control group; IQR, Interquartile Range; NR, not report; M1: EFS, event-free survival; M2: OS, overall survival; M3: MRD, minimal residual disease response; M4: Relapse; M5: AE, adverse events; M6: CR, complete remission. MRD, response rate was defined by the incidence of negative MRD.

### 3.2 Quality assessment

The methodological quality of the two RCTs included is summarized in (Supplementary Figures S2, S3). Both studies reported acceptable methods of randomization. However, they didn’t explicitly mention whether or not allocation schemes were hidden. Among the single-arm studies, three included only nine patients (Schlegel et al., 2014; Ampatzidou et al., 2020; Horibe et al., 2020), and one included 13 patients (Pawinska-Wasikowska et al., 2022) and one included 15 patients (Fuster et al., 2020). A too small sample size affects the consistency of the results, the collection of expected data, the appropriateness of endpoint indicators to reflect the purpose of the study, the objectivity of endpoint evaluation, and whether the sample size has been estimated. In all other studies, the number of patients lost to follow-up was acceptable (<20%) (Supplementary Figure S4). The quality for clinical trials enrolled was moderate to high.

Supplementary Figure S5 shows the sensitivity analyses of all eligible studies, and Supplementary Figure S6 shows corresponding forest plots. Funnel plots were used to assess the potential publication bias in the reporting of MRD (Supplementary Figure S7). The pooled results showed no evidence of significant publication bias. In addition, Egger’s test was used to evaluate the publication bias in the reporting of MRD. It was found that *p* = .576 >.05, corroborating that there was no significant publication bias (Supplementary Figure S8).

### 3.3 Efficacy

#### 3.3.1 CR

**FIGURE 2:**
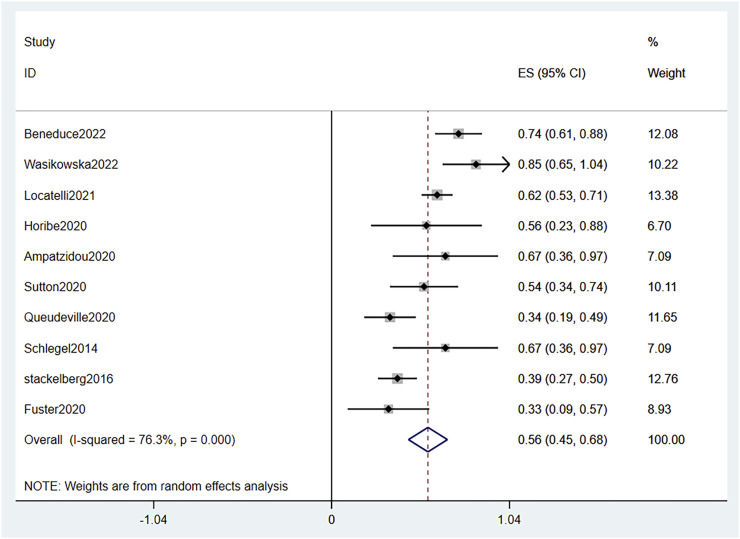
Forest plot of CR. CR, complete response; 95% CI, 95% confidence interval; ES: Effect Size. The size of the rectangle at the center of the horizontal bar is proportional to the weight of the given study. The diamond at the bottom indicates the pooled ES (only single-arm studies have CR data).

In total, 336 patients from 10 single-arm studies were enrolled, and 183 patients achieved CR. The effect size (ES) of the CR varies from .33 to .85, with a pooled ES Size of .56 (95% CI .54–.68) according to the random effects model. We observed substantial heterogeneity between the studies (I^2^ = 76.3%, *p* = .000) (Figure 2). Therefore, we conducted a sensitivity analysis. The results showed that the sensitivity was low and the results were relatively stable.

To investigated whether previous blast load affected the CR rate of patients receiving blinatumomab treatment, we compared the ES between patients with high blast percentages in the BM (≥50%) and those with low blast percentages in the BM (<50%) in three sing-arm studies (Schlegel et al., 2014; von Stackelberg et al., 2016; Pawinska-Wasikowska et al., 2022). The pooled ES of patients with BM blast <50% (.61, 95% CI -.14–1.37) was higher than that of patients with BM blast ≥50% (-.64, 95% CI -1.21–.08) (Supplementary Figure S6).

#### 3.3.2 OS

**FIGURE 3:**
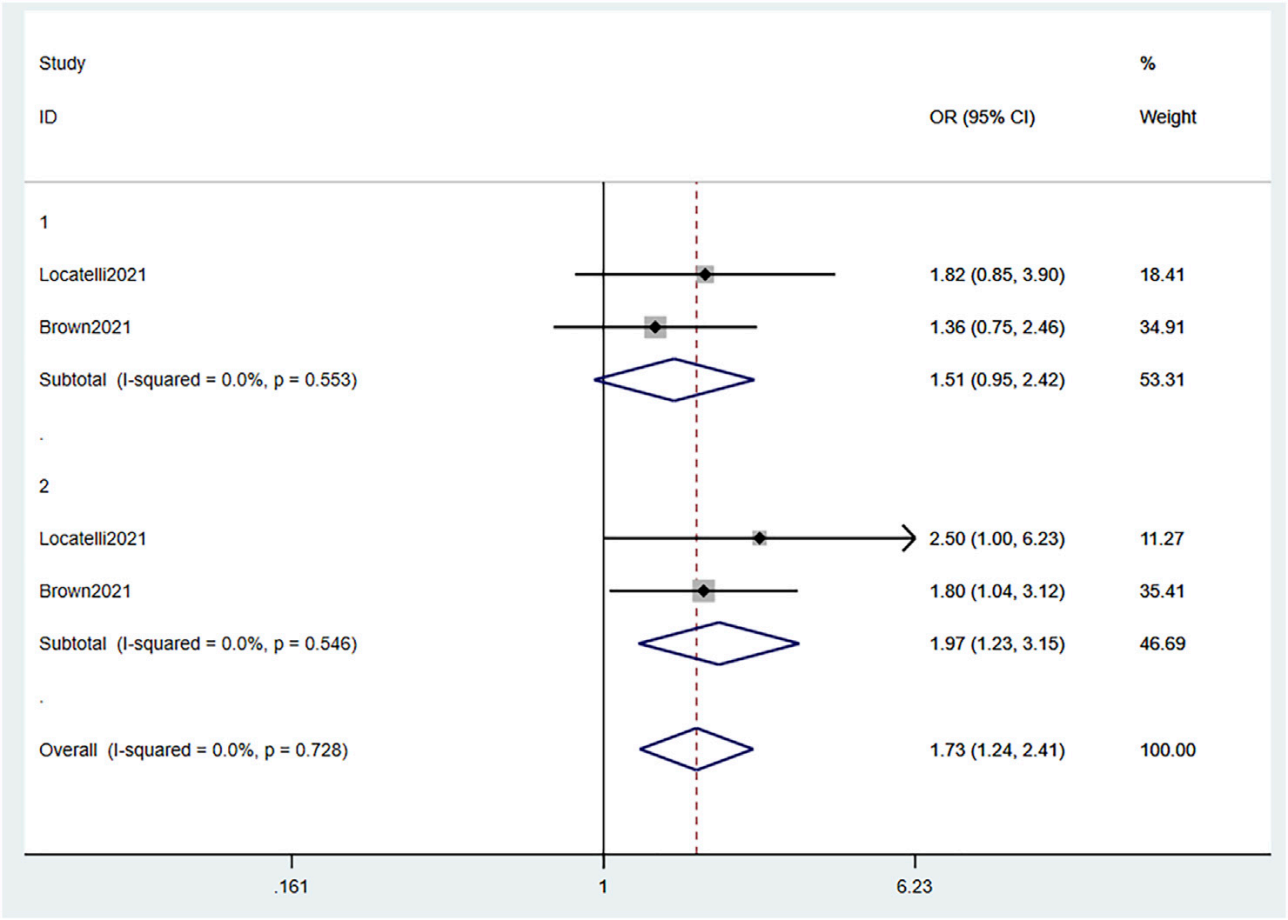
Forest plot of OS (RCTs). OS, overall survival; RCTs, randomized clinical trials; 95% CI, 95% confidence interval; OR: odds ratio; 1, 1-year; 2, 2-year. The size of the rectangle at the center of the horizontal bar is proportional to the weight of the given study. The diamond at the bottom indicates the pooled OR.

**FIGURE 4:**
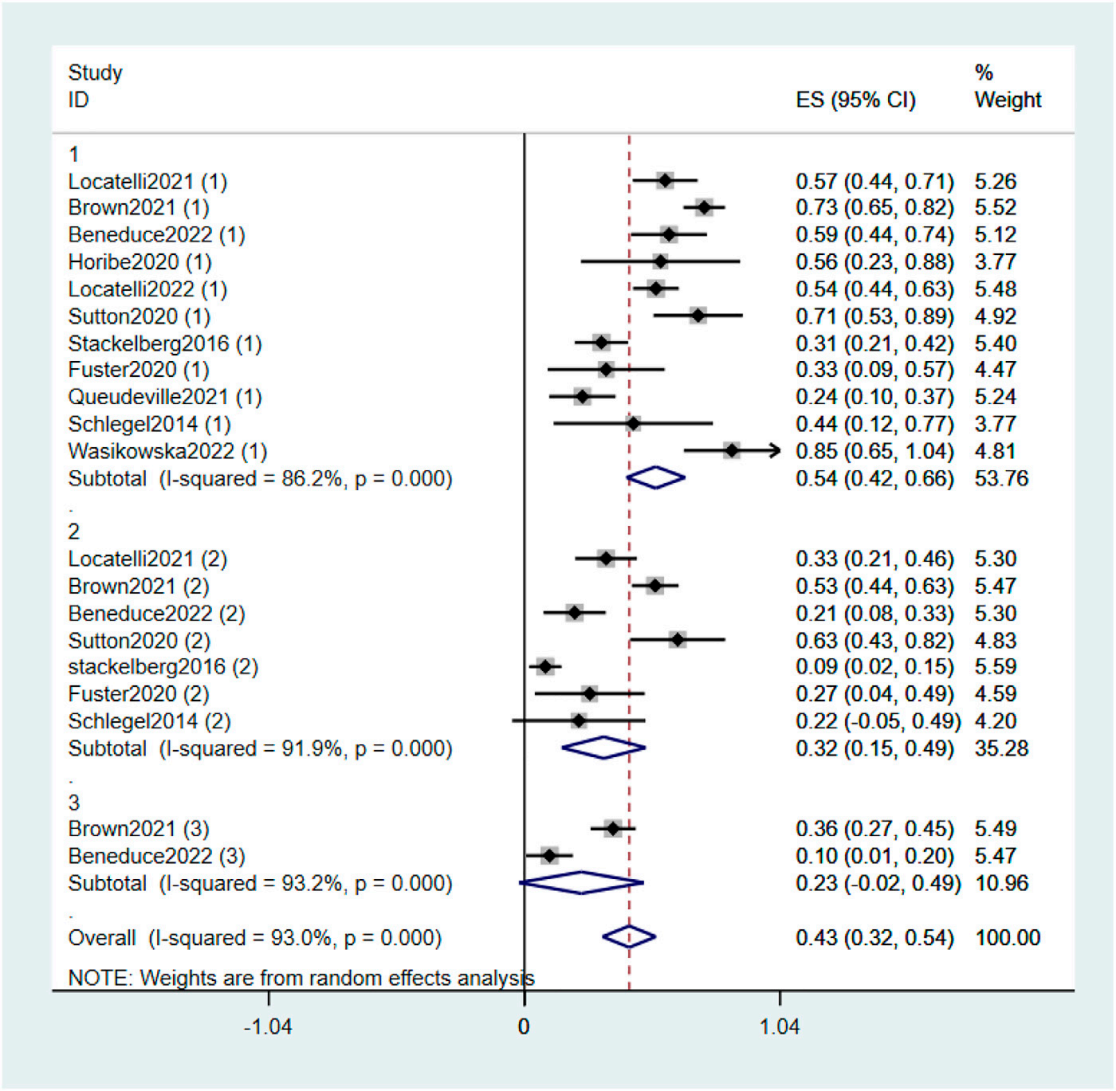
Forest plot of OS (single-arm studies). OS, overall survival; 95% CI, 95% confidence interval; ES: Effect Size; 1, 1-year; 2, 2-year; 3, 3-year. The size of the rectangle at the center of the horizontal bar is proportional to the weight of the given study. The diamond at the bottom indicates the pooled ES.

Combined data of the two RCTs revealed that 1-year OS was not statistically significantly different between the blinatumomab treatment group and the Chemotherapy treatment group (OR 1.51, 95% CI .95–2.42 I^2^ = .0% *p* = .553), whereas 2-year OS was (OR 1.97, 95% CI 1.23–3.15 I^2^ = .0% *p* = .546). The all-time OS based on the combined data was significantly different between the groups (OR 1.73, 95% CI 1.24–2.41 I^2^ = .0% *p* = .728) (Figure 3). This indicated that blinatumomab can improve OS in children with R/R B-ALL compared to chemotherapy. The patients in the experimental arm in the two RCTs and the nine single-arm studies were evaluated for OS after blinatumomab treatment (Schlegel et al., 2014; von Stackelberg et al., 2016; Fuster et al., 2020; Horibe et al., 2020; Queudeville et al., 2021; Sutton et al., 2021; Beneduce et al., 2022; Locatelli et al., 2022; Pawinska-Wasikowska et al., 2022). The pooled ORs of OS was .43 (95% CI .32–.54) with substantial heterogeneity observed (I^2^ = 93.0%, *p* = .0000) (Figure 4). Sensitivity analysis shows that the sensitivity was low, and the results were relatively stable.

#### 3.3.3 EFS

**FIGURE 5:**
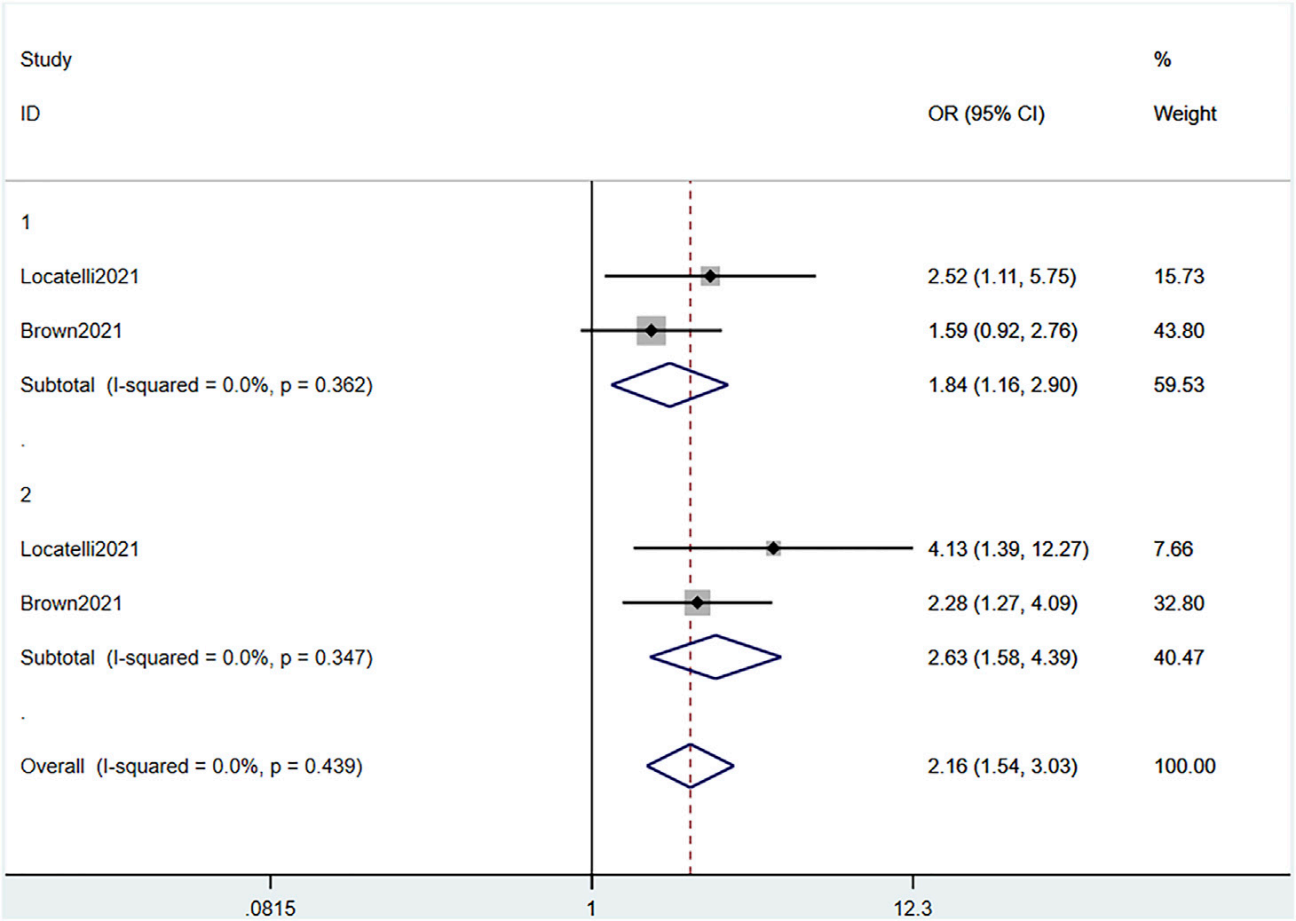
Forest plot of EFS (RCTs). EFS, event-free survival; RCTs, randomized clinical trials; OR: odds ratio; 1, 1-year; 2, 2-year; 3, 3-year. The size of the rectangle at the center of the horizontal bar is proportional to the weight of the given study. The diamond at the bottom indicates the pooled OR.

**FIGURE 6:**
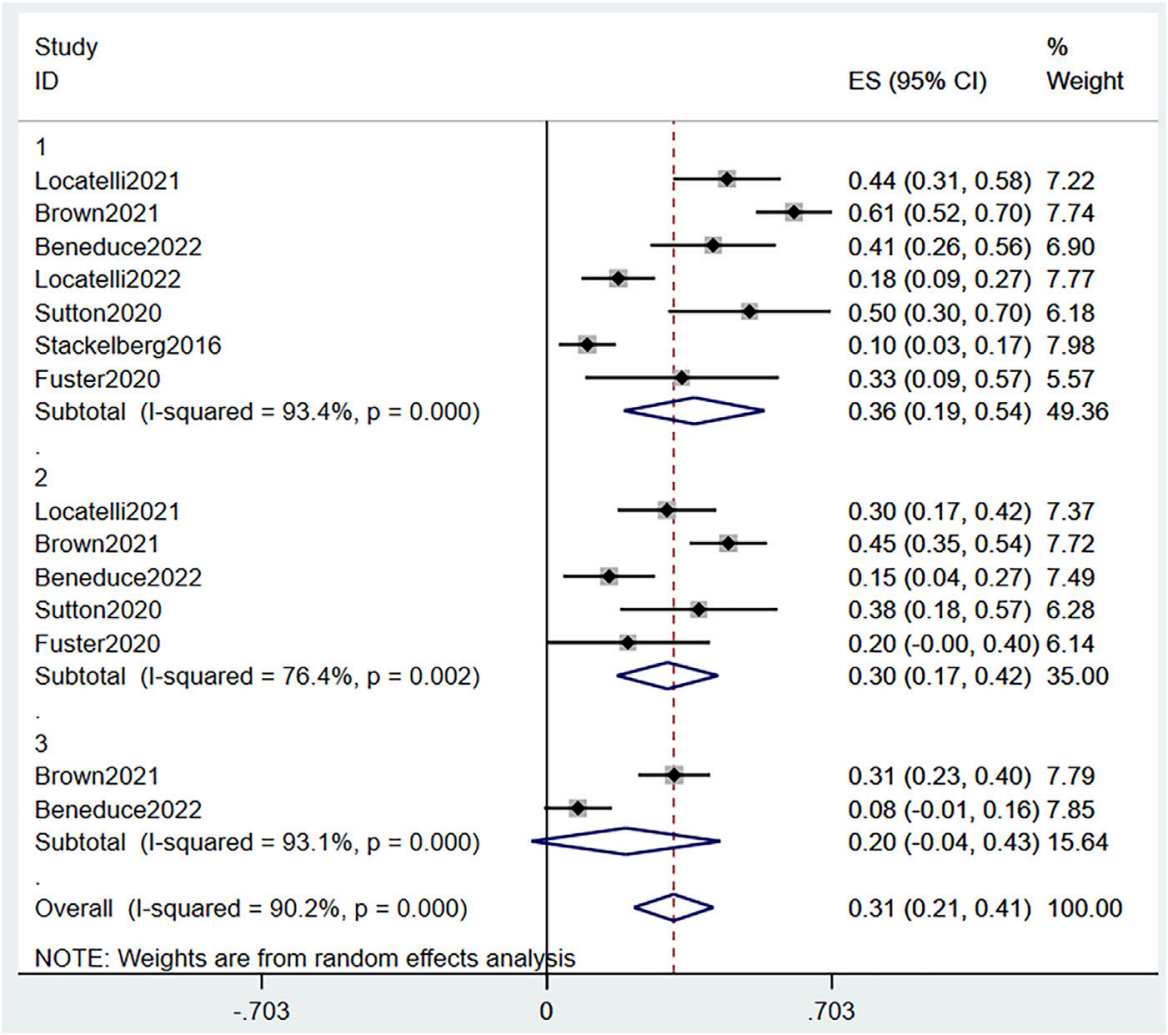
Forest plot of EFS (single-arm studies). EFS, event-free survival; 95% CI, 95% confidence interval; ES: Effect Size; 1, 1-year; 2, 2-year; 3, 3-year. The size of the rectangle at the center of the horizontal bar is proportional to the weight of the given study. The diamond at the bottom indicates the pooled ES.

Based on combined data of the two RCTs, 1- and 2-years EFS were both significantly different between the blinatumomab and the chemotherapy groups (OR 1.84, 95% CI 1.16–2.90 I^2^ = .0% *p* = .362) and (OR 2.63, 95% CI 1.58–4.39 I^2^ = .0% *p* = .347), respectively All-time EFS based on the combined data also showed a significant difference between the groups (OR 2.16, 95% CI 1.54–3.03 I^2^ = .0% *p* = .439) (Figure 5). EFS was significantly prolonged after blinatumomab compared with chemotherapy. The pooled EFS rate in the experimental groups of the two RCTs and five single-arm studies (von Stackelberg et al., 2016; Fuster et al., 2020; Sutton et al., 2021; Beneduce et al., 2022; Locatelli et al., 2022) was .31 (95% CI .21–.41), with substantial heterogeneity observed (I^2^ = 90.2%, *p* = .0000) (Figure 6). Sensitivity analysis showed that the sensitivity was low, and the results were relatively stable.

#### 3.3.4 MRD

**FIGURE 7:**
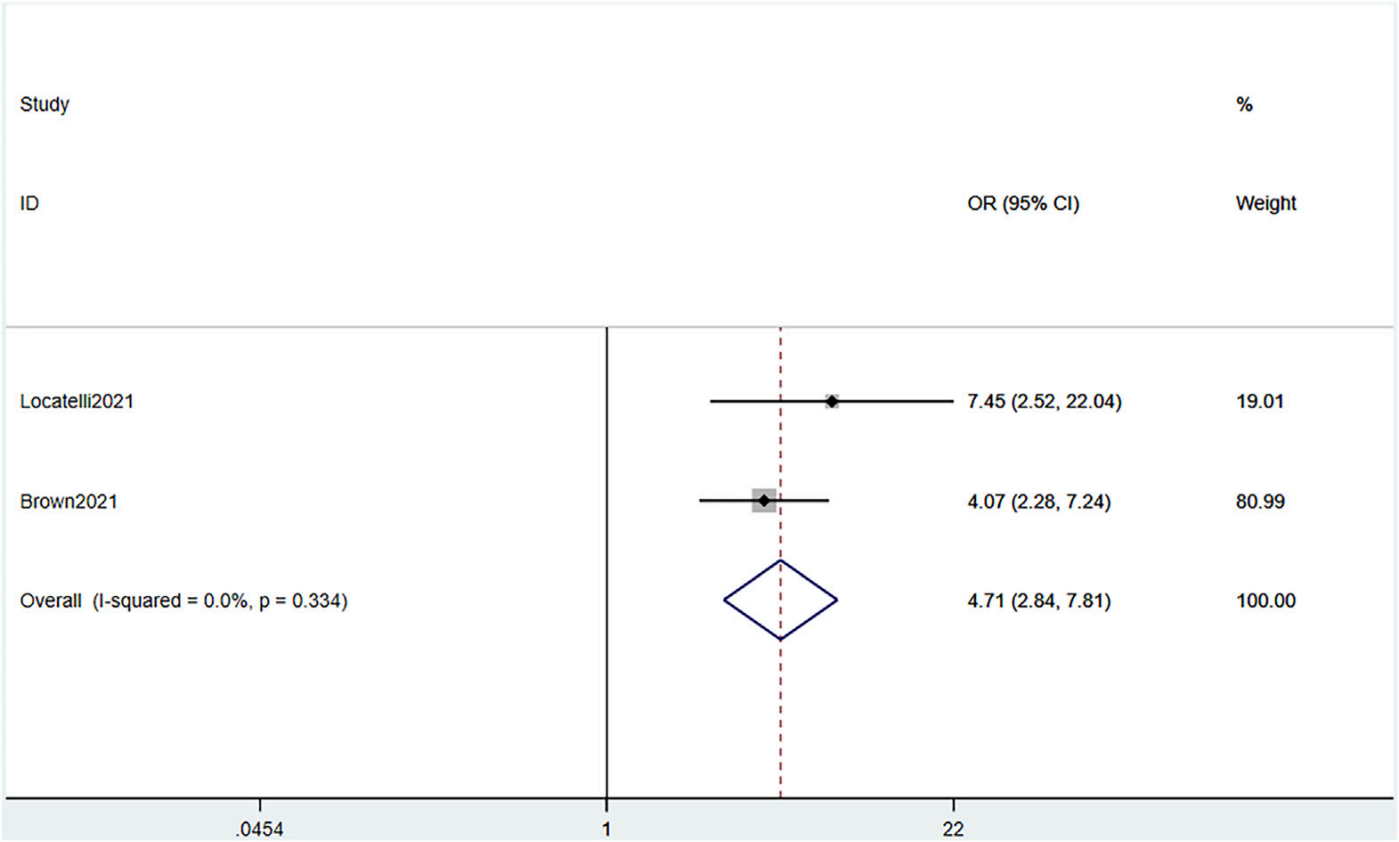
Forest plot of MRD (RCTs). MRD, minimal residual disease; RCTs, randomized clinical trials; 95% confidence interval; OR: odds ratio. The size of the rectangle at the center of the horizontal bar is proportional to the weight of the given study. The diamond at the bottom indicates the pooled OR.

**FIGURE 8:**
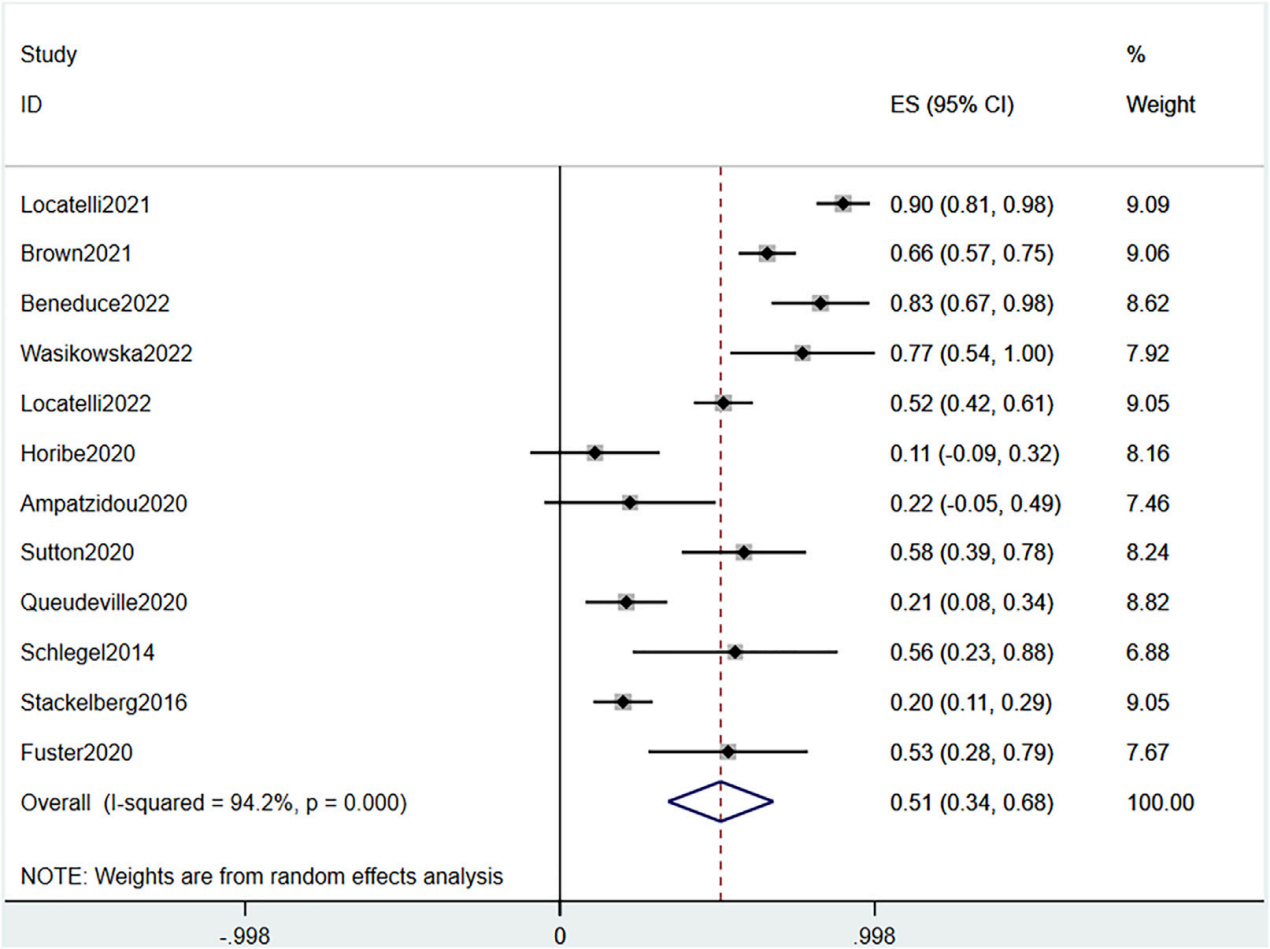
Forest plot of MRD (single-arm studies). MRD, minimal residual disease; 95% CI, 95% confidence interval; ES: Effect Size. The size of the rectangle at the center of the horizontal bar is proportional to the weight of the given study. The diamond at the bottom indicates the pooled ES.

Combined data of the two RCTs revealed a significant difference in MRD response between the blinatumomab and the chemotherapy groups (OR 4.71, 95% CI 2.84–7.81 I^2^ = .0% *p* = .334) (Figure 7). The total of 692 patients from all 12 studies were evaluated for MRD response after blinatumomab treatment. The pooled MRD response rate was .51 (95% CI .34–.68), with substantial heterogeneity observed (I^2^ = 94.2%, *p* = .0000) (Figure 8). Sensitivity analysis showed that the sensitivity was low, and the results were relatively stable.

#### 3.3.5 Allo-HSCT

In the two RCTs, there were 82 patients in the chemotherapy group, and 122 patients in the blinatumomab treatment group received allo-HSCT any time after the first blinatumomab infusion. Analysis of the combined data showed that there was a significant difference in allo-HSCT between the two groups (OR 3.24, 95% CI 1.96–5.35 I^2^ = .0% *p* = .932). When combining all studies, a total of 303 patients underwent allo-HSCT after blinatumomab treatment (OR .62, 95% CI .50–.74), with substantial heterogeneity observed (I^2^ = 87.4%, *p* = .0000). Sensitivity analysis showed that the sensitivity was low, and the results were relatively stable.

### 3.4 Safety

#### 3.4.1 AEs

**FIGURE 9:**
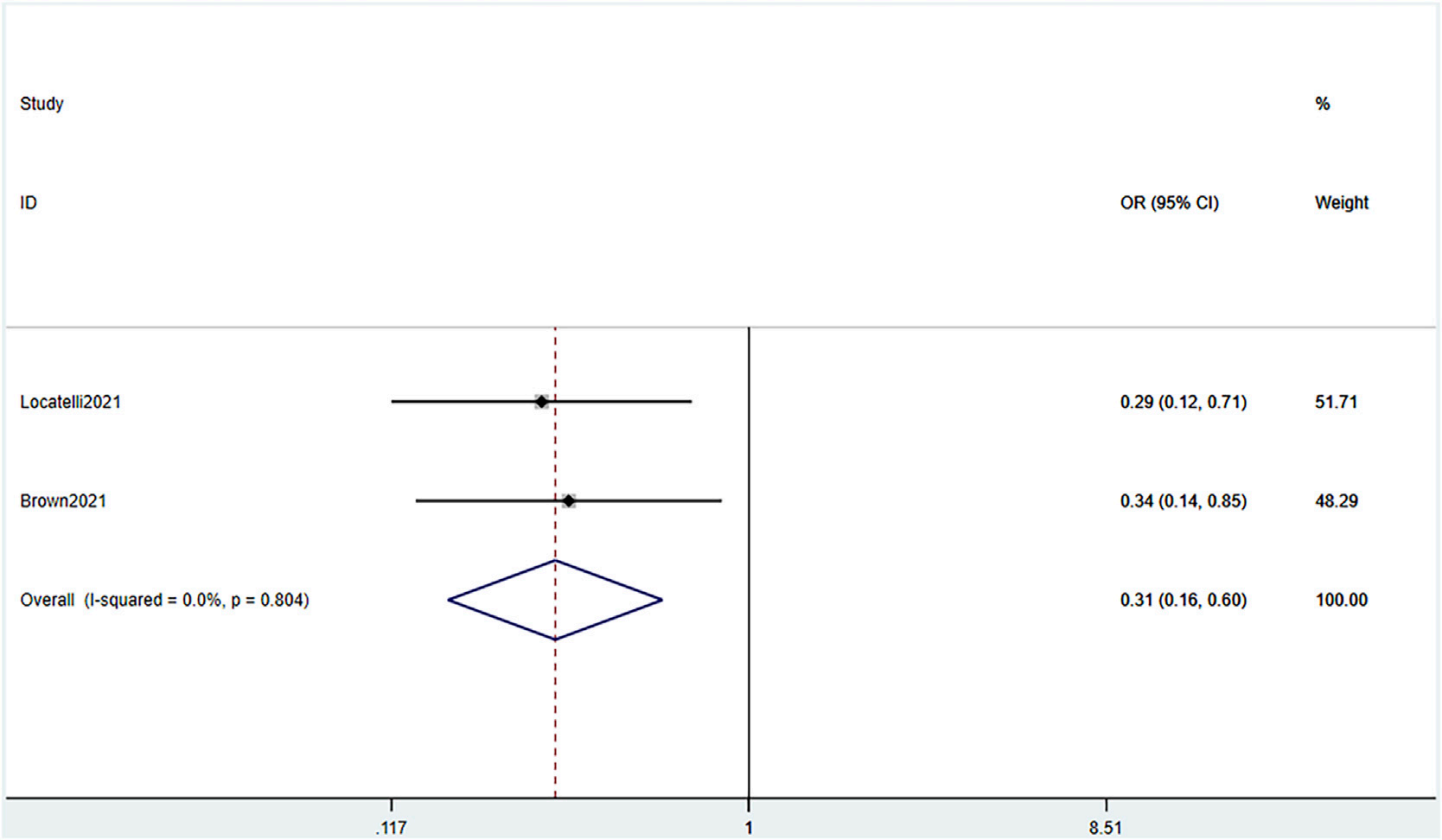
Forest plot of AE (RCTs). AE, adverse effects; RCTs, randomized clinical trials; 95% confidence interval; OR: odds ratio. The size of the rectangle at the center of the horizontal bar is proportional to the weight of the given study. The diamond at the bottom indicates the pooled OR.

**FIGURE 10:**
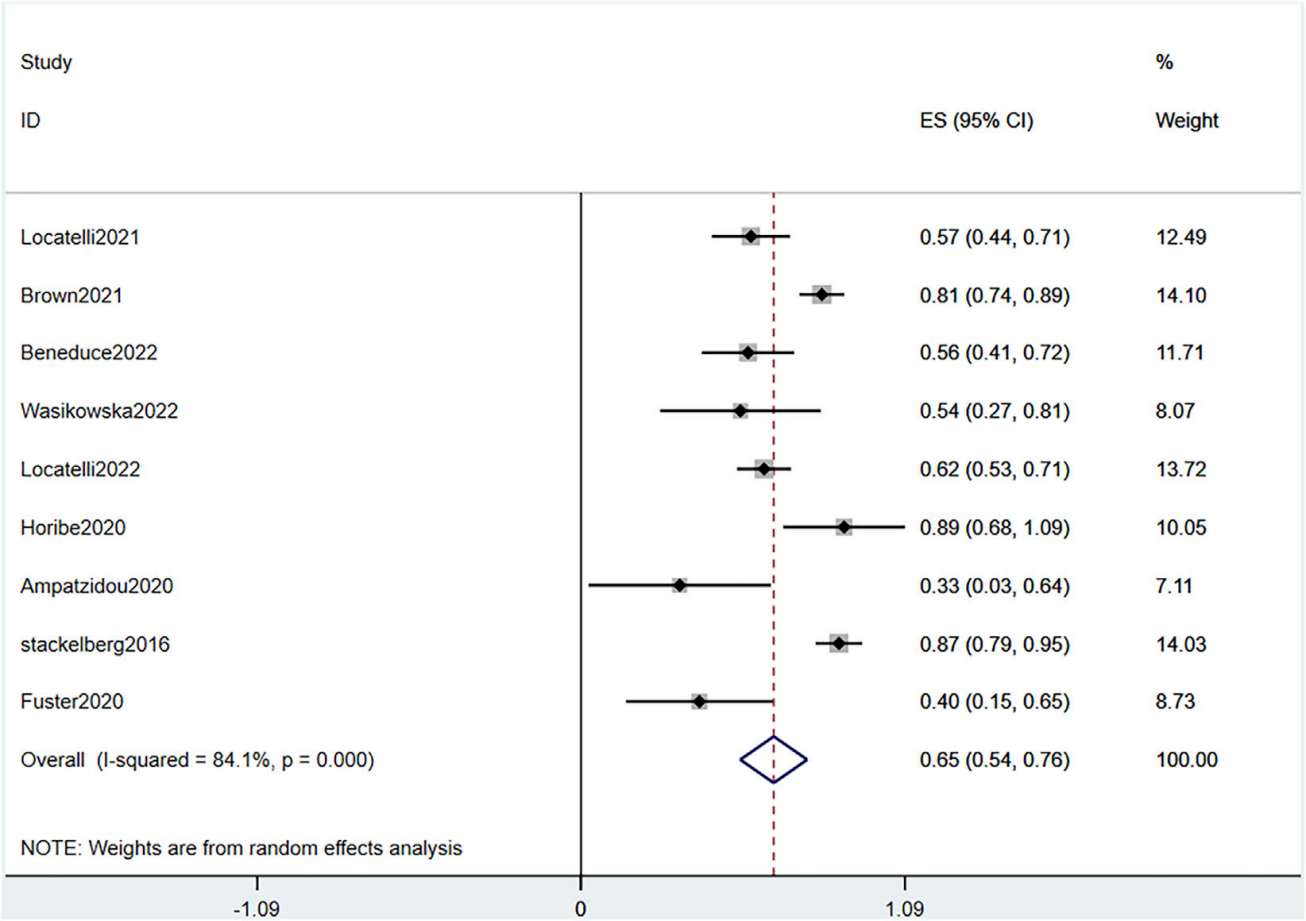
Forest plot of AE (single-arm studies). AE, adverse effects; 95% CI, 95% confidence interval; ES: Effect Size. The size of the rectangle at the center of the horizontal bar is proportional to the weight of the given study. The diamond at the bottom indicates the pooled ES.

AEs were graded according to the National Cancer Institute Common Terminology Criteria for Adverse Events version 4.03. Because the grades and specific AEs were inconsistent across the studies and the sample sizes varied, we assessed the incidence of grade 3 or higher AEs. Analysis of combined data of the two RCTs showed a significant difference of AEs between the blinatumomab and the chemotherapy groups (OR .31, 95% CI .16–.60 I^2^ = .0% *p* = .804) (Figure 9). The pooled AE rate in the experimental groups of the two RCTs and seven single-arm studies (von Stackelberg et al., 2016; Ampatzidou et al., 2020; Fuster et al., 2020; Horibe et al., 2020; Beneduce et al., 2022; Locatelli et al., 2022; Pawinska-Wasikowska et al., 2022) was .65 (95% CI: .54–.76) with substantial heterogeneity observed (I^2^ = 84.1%, *p* = .0000) (Figure 10). Sensitivity analysis showed that the sensitivity was low, and the results were relatively stable. The most common AEs were cytokine release syndrome (CRS), neutropenia, deaths and neurological events such as pyrexia, anemia, nausea, and headache. In total, 75 CRS were reported in 467 patients; 100 grade 3 or higher neutropenia were reported in 295 patients; 119 deaths were reported in 495 patients; the frequency of CRS, grade 3 or higher neutropenia, death during blinatumomab therapy was 16%, 33.8%, 24% respectively. In two RCTs, the incidence of grade 3 or higher neutropenia and deaths in the blinatumomab group was 23% and 8%, respectively. The incidence of grade 3 or higher neutropenia and deaths in the consolidation chemotherapy group was 47.2% and 21.6%, respectively.

#### 3.4.2 Relapse

The combined RCT study data showed no significant difference in the relapse rate between the blinatumomab and chemotherapy groups (OR .69, 95% CI .43–1.09 I^2^ = 86.6% *p* = .006). The pooled relapse rate in experimental groups of the two RCTs and four single-arm studies (Ampatzidou et al., 2020; Fuster et al., 2020; Sutton et al., 2021; Beneduce et al., 2022) was .32 (95% CI .27–.38 I^2^ 32.0%, *p* = .196).

## 4 Discussion

Our analysis validated the efficacy and safety of blinatumomab in children with R/R B -ALL based on the data from RCTs and single-arm studies. We evaluated the therapeutic effects of blinatumomab in terms of CR, OS, EFS, MRD, allo-HSCT, AEs, relapse, and safety using data from two RCT studies (in comparison with chemotherapy) and 10 single-arm studies. The pooled CR rate after blinatumomab treatment was .56, indicating that blinatumomab is effective in the treatment of R/R B-ALL in children. OS and EFS were significantly prolonged after blinatumomab as compared to chemotherapy, suggesting that blinatumomab treatment can prolong the OS period of children with R/R B-ALL. Blinatumomab was more effective in eliminating MRD than chemotherapy (OR 4.71, 95% CI 2.84–7.81 I^2^ = .0% *p* = .334). The pooled MRD response rate was .51, corroborating that blinatumomab can eliminate MRD. Allo-HSCT is critical for many patients with R/R hematological malignancies. Leukemia patients with CR of their primary disease before allo-HSCT are less likely to relapse after transplantation (Krauter et al., 2011; Bastos-Oreiro et al., 2014). In our analysis, more patients received allo-HSCT after blinatumomab than after chemotherapy (OR 3.24, 95% CI 1.96–5.35 I^2^ = .0% *p* = .932). In the single-arm studies, 303 patients underwent allo-HSCT after blinatumomab treatment (OR 95% CI .50–.74), which demonstrated that blinatumomab can be used as a bridge therapy to HSCT. In addition, ALL patients with low BM blast levels achieved higher CR rates than patients with high BM blast levels, implying that higher tumor burden is associated with poorer responses. These results suggest that chemotherapy pretreatment is required to reduce tumor burden prior to blinatumomab treatment in R/R B-ALL patients.

In our analysis, the main AEs after blinatumomab treatment were CRS and neurological events. The frequency of CRS in anti-CD19 Chimeric Antigen Receptor T cell Immunotherapy therapy was as high as 93% in a phase I/II trial (Gardner et al., 2017). However, in our study, only 75 of 467 patients (16%) experienced CRS. This may be because of the use of stepped doses of blinatumomab in the treatment and because all patients were treated with steroids before blinatumomab. Temporary treatment discontinuation is the most commonly implemented strategy to address blinatumomab-related toxicity. The proportion of patients experiencing CRS and neurotoxic AEs varied across studies, which may be because of the variable levels of BM blasts at baseline. The studies suggest that blinatumomab has a good safety profile for R/R B-ALL, especially in cases with limited leukemia burden. A meta-analysis of the safety of blinatumomab in childhood leukemia by (Marrapodi et al., 2022) demonstrated that compared with chemotherapy, blinatumomab was associated with grade 3 or higher AEs (risk ratio, .79, 95% CI .67–.93) and CRS risk (risk ratio 8.37, 95% CI .27–260.97). This is roughly consistent with our data, but our study included a larger base, we also analyzed relapse, which is crucial in the treatment of leukemia.

Blinatumomab treatment is aimed at creating the conditions for stem cell transplantation necessary to achieve durable remission. A higher proportion of trial participants who received blinatumomab compared with conventional chemotherapy were able to go on to transplant, likely because blinatumomab treatment resulted in higher rates of MRD negativity and lower rates of AEs. The lower risk of disease recurrence in blinatumomab-treated patients is consistent with data showing MRD remission before allo-HSCT to improve posttransplant outcomes in children with ALL (Bader et al., 2009; Ruggeri et al., 2012). Thus, blinatumomab monotherapy represents a valuable consolidation therapy that appears more effective than conventional chemotherapy before transplantation for this patient population.

This study was the first meta-analysis to evaluate the efficacy and safety of blinatumomab in children with R/R B-ALL. However, the study had limitations. First, the sample size was relatively small, with only two RCTs, and three single-arm trials involving only nine patients. Second, the random-effects model we used in this study minimizes inherent variance. Third, in the RCT of Brown et al., some patients were older than 18 years and it was not possible to select only events that occurred in the pediatric cohort. Finally, because of the limited number of patients, we did not conduct genetic analyses.

## 5 Conclusion

Our meta-analysis showed that blinatumomab provides significant benefits in children with R/R B-ALL. We found that a lower tumor burden was beneficial to the therapeutic effect. As for AEs, serious CRS and neurological events were infrequent. We conclude that blinatumomab is a safe and feasible treatment for children with R/R B-ALL and should be initiated as soon as possible. Future multi-center, high-quality, and larger-sample clinical studies will be required to validate our findings.

## Data Availability

The original contributions presented in the study are included in the article/Supplementary Material, further inquiries can be directed to the corresponding authors.
