# Supplementary material for: Efficacy and safety of blinatumomab in children with relapsed/refractory B cell acute lymphoblastic leukemia: A systematic review and meta-analysis
Source: Front Pharmacol. 2023 Jan 10;13:1032664. doi: 10.3389/fphar.2022.1032664 (PMC9871389; doi:10.3389/fphar.2022.1032664)
Supplement: Supplementary file 1 [file DataSheet1.docx]

**Supplementary material**

**Supplementary Data 1:** PubMed search history

| No | Query |
| --- | --- |
| #1 | "blinatumomab" [Supplementary Concept] |
| #2 | ((((blinatumomab[Title/Abstract]) OR (MT-103 antibody[Title/Abstract])) OR (antibody MT-103[Title/Abstract])) OR (Blincyto[Title/Abstract])) OR (MEDI-538[Title/Abstract]) |
| #3 | #1 OR #2 |
| #4 | "Precursor Cell Lymphoblastic Leukemia-Lymphoma"[Mesh] |
| #5 | ((((((((((((((((((((((((((((((((((Precursor Cell Lymphoblastic Leukemia-Lymphoma[Title/Abstract]) OR (Precursor Cell Lymphoblastic Leukemia Lymphoma[Title/Abstract])) OR (Leukemia, Acute Lymphoblastic[Title/Abstract])) OR (Acute Lymphoblastic Leukemia[Title/Abstract])) OR (Leukemia, Lymphoblastic[Title/Abstract])) OR (Leukemia, Lymphoblastic, Acute[Title/Abstract])) OR (Leukemia, Lymphocytic, Acute[Title/Abstract])) OR (Lymphoblastic Leukemia[Title/Abstract])) OR (Lymphoblastic Leukemia, Acute[Title/Abstract])) OR (Lymphoblastic Lymphoma[Title/Abstract])) OR (Lymphocytic Leukemia, Acute[Title/Abstract])) OR (Acute Lymphocytic Leukemia[Title/Abstract])) OR (Leukemia, Acute Lymphocytic[Title/Abstract])) OR (Lymphoma, Lymphoblastic[Title/Abstract])) OR (Acute Lymphoid Leukemia[Title/Abstract])) OR (Leukemia, Acute Lymphoid[Title/Abstract])) OR (Lymphoid Leukemia, Acute[Title/Abstract])) OR (Leukemia, Lymphoid, Acute[Title/Abstract])) OR (Leukemia, Lymphocytic, Acute, L1[Title/Abstract])) OR (Lymphocytic Leukemia, L1[Title/Abstract])) OR (L1 Lymphocytic Leukemia[Title/Abstract])) OR (Leukemia, L1 Lymphocytic[Title/Abstract])) OR (Lymphoblastic Leukemia, Acute, Childhood[Title/Abstract])) OR (Lymphoblastic Leukemia, Acute, L1[Title/Abstract])) OR (ALL, Childhood[Title/Abstract])) OR (Childhood ALL[Title/Abstract])) OR (Leukemia, Lymphoblastic, Acute, L1[Title/Abstract])) OR (Leukemia, Lymphocytic, Acute, L2[Title/Abstract])) OR (Lymphocytic Leukemia, L2[Title/Abstract])) OR (L2 Lymphocytic Leukemia[Title/Abstract])) OR (Leukemia, L2 Lymphocytic[Title/Abstract])) OR (Lymphoblastic Leukemia, Acute, Adult[Title/Abstract])) OR (Lymphoblastic Leukemia, Acute, L2[Title/Abstract])) OR (Leukemia, Lymphoblastic, Acute, L2[Title/Abstract])) OR (Leukemia, Lymphoblastic, Acute, Philadelphia-Positive[Title/Abstract]) |
| #6 | #4 OR #5 |
| #7 | "Child"[Mesh] |
| #8 | (child[Title/Abstract]) OR (children[Title/Abstract]) |
| #9 | "Pediatrics"[Mesh] |
| #10 | Pediatrics[Title/Abstract] |
| #11 | #7 OR #8 OR #9 OR #10 |
| #12 | #3 AND #6 AND #11 |

|  |  |
| --- | --- |


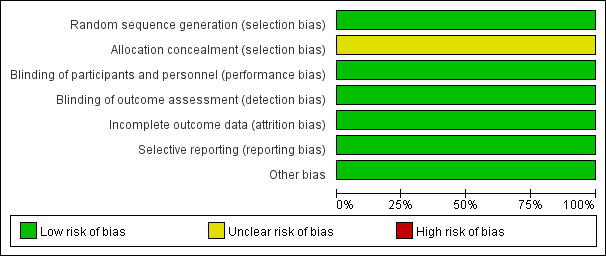


**Supplementary Data 2:** Risk of bias graph (RCTs). RCTs, randomized clinical trials.


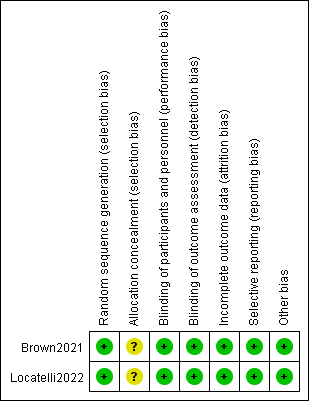


**Supplementary Data 3:** Risk of bias summary (RCTs). RCTs, randomized clinical trials.

| \| study \| \| --- \| | The purpose of the research is clearly stated | Consistency of enrolled patients | Collection of expected data | Endpoints that appropriately reflect the purpose of the study | The objectivity of endpoint evaluation | Adequate follow-up time | Loss to follow-up rate is less than 5% | Whether the sample size was estimated | total |
| --- | --- | --- | --- | --- | --- | --- | --- | --- | --- | --- |
| Beneduce2022 | 2 | 1 | 2 | 2 | 2 | 2 | 2 | 1 | 14 |
| Wasikowska2022 | 2 | 1 | 1 | 1 | 1 | 2 | 2 | 1 | 12 |
| Locatelli2021 | 2 | 2 | 2 | 2 | 2 | 1 | 2 | 2 | 15 |
| Horibe2020 | 2 | 1 | 1 | 1 | 1 | 2 | 2 | 1 | 12 |
| Ampatzidou2020 | 2 | 1 | 1 | 1 | 1 | 1 | 2 | 1 | 10 |
| Sutton2020 | 2 | 1 | 2 | 2 | 2 | 2 | 2 | 1 | 14 |
| Queudeville2020 | 2 | 1 | 2 | 2 | 2 | 2 | 2 | 2 | 15 |
| Schlegel2014 | 2 | 1 | 1 | 1 | 1 | 2 | 2 | 1 | 11 |
| Stackelberg2016 | 2 | 2 | 2 | 2 | 2 | 2 | 2 | 2 | 16 |
| Fuster2020 | 2 | 1 | 1 | 1 | 1 | 1 | 2 | 1 | 10 |

Supplementary Data 4**:** Risk of bias in single-arm studies

**Supplementary Data 5:** Sensitivity analysis. **(A)** Sensitivity analysis of CR; **(B)** Sensitivity analysis of OS (single-arm studies); **(C)** Sensitivity analysis of EFS (single-arm studies); **(D)** Sensitivity analysis of MRD (single-arm studies); **(E)** Sensitivity analysis of allo-HSCT (single-arm studies); **(F)** Sensitivity analysis of AE (single-arm studies); **(G)** Sensitivity analysis of relapse (single-arm studies). CR, complete response; OS, overall survival; EFS, event-free survival; MRD, minimal residual disease; allo-HSCT, allogeneic hematopoietic stem cell transplantation; AE, adverse effects.


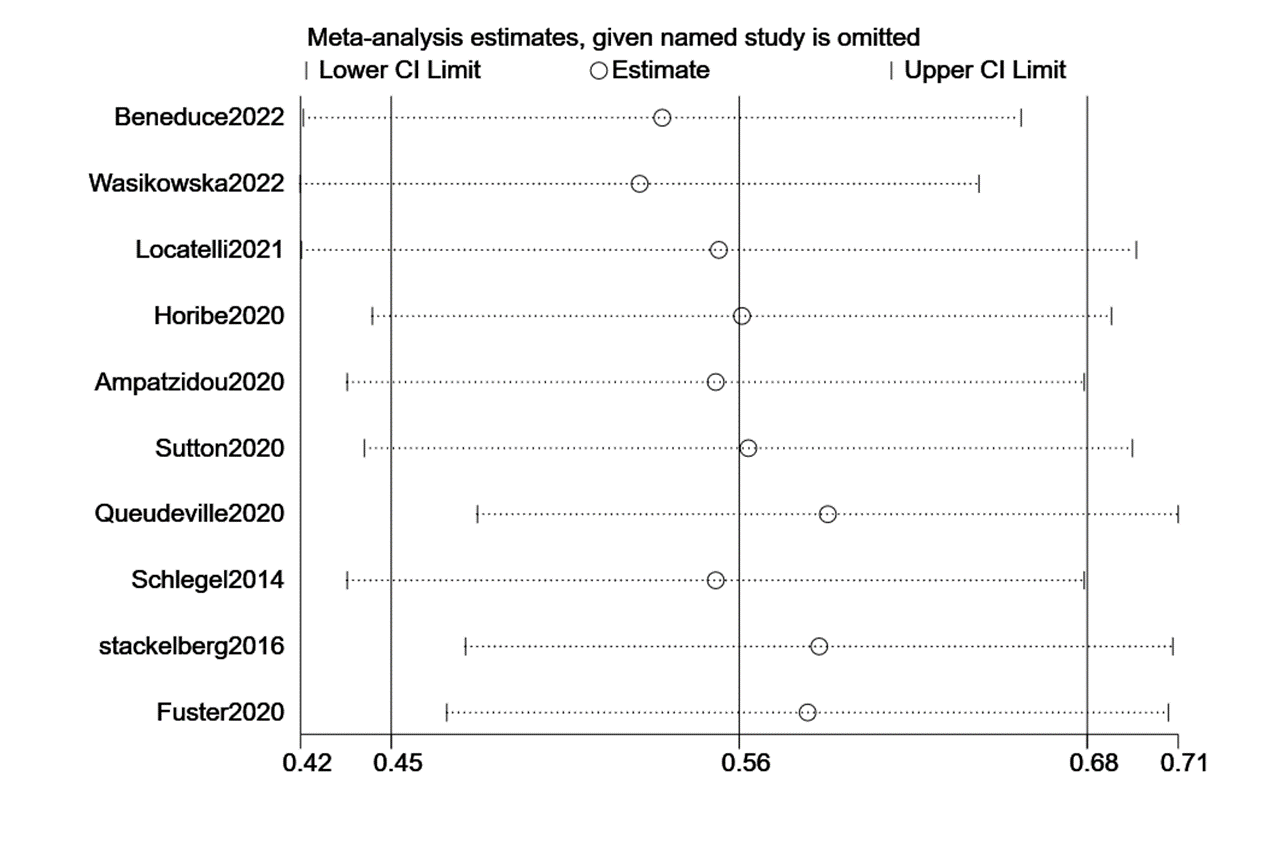


**A:** Sensitivity analysis of CR. CR, complete response.


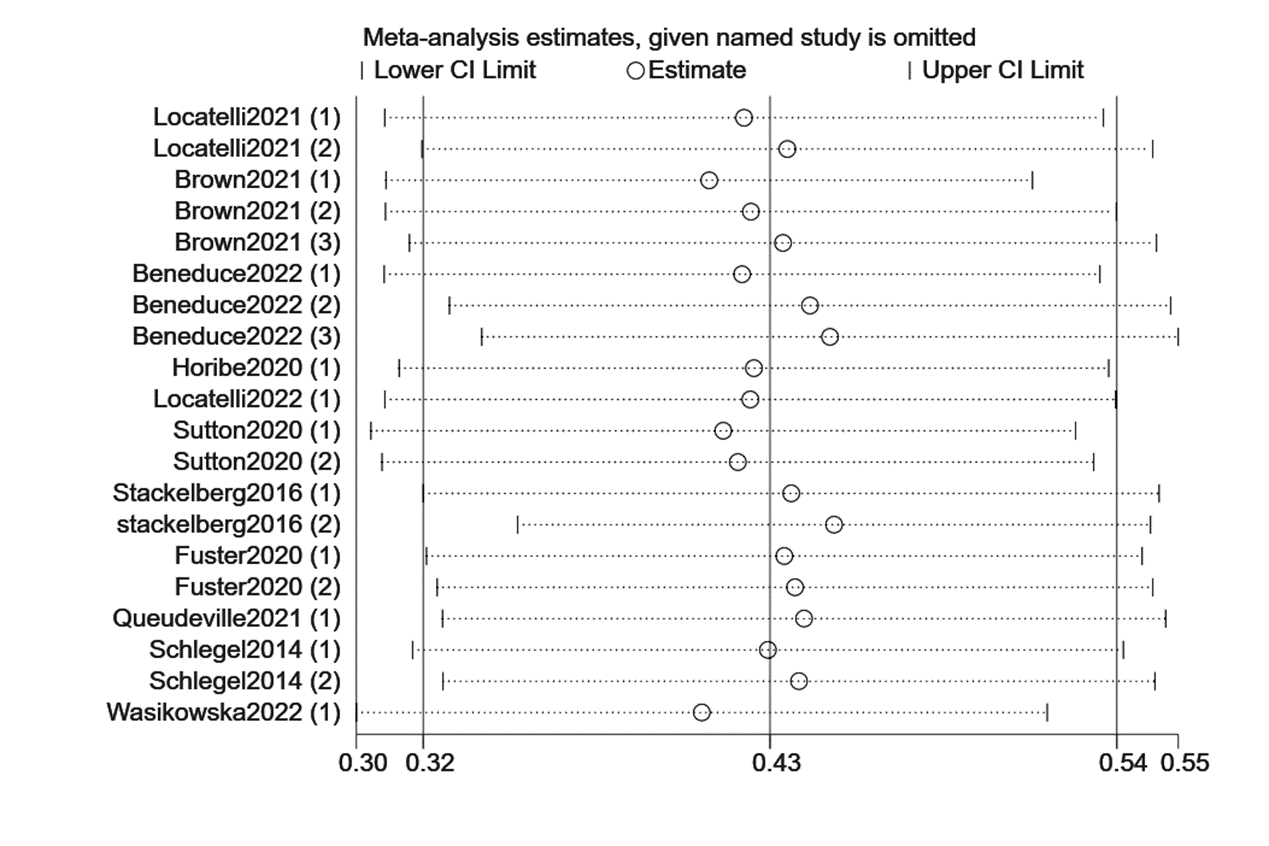


**B:** Sensitivity analysis of OS (single-arm studies). OS, overall survival; 1, first year; 2, second year; 3, third year.


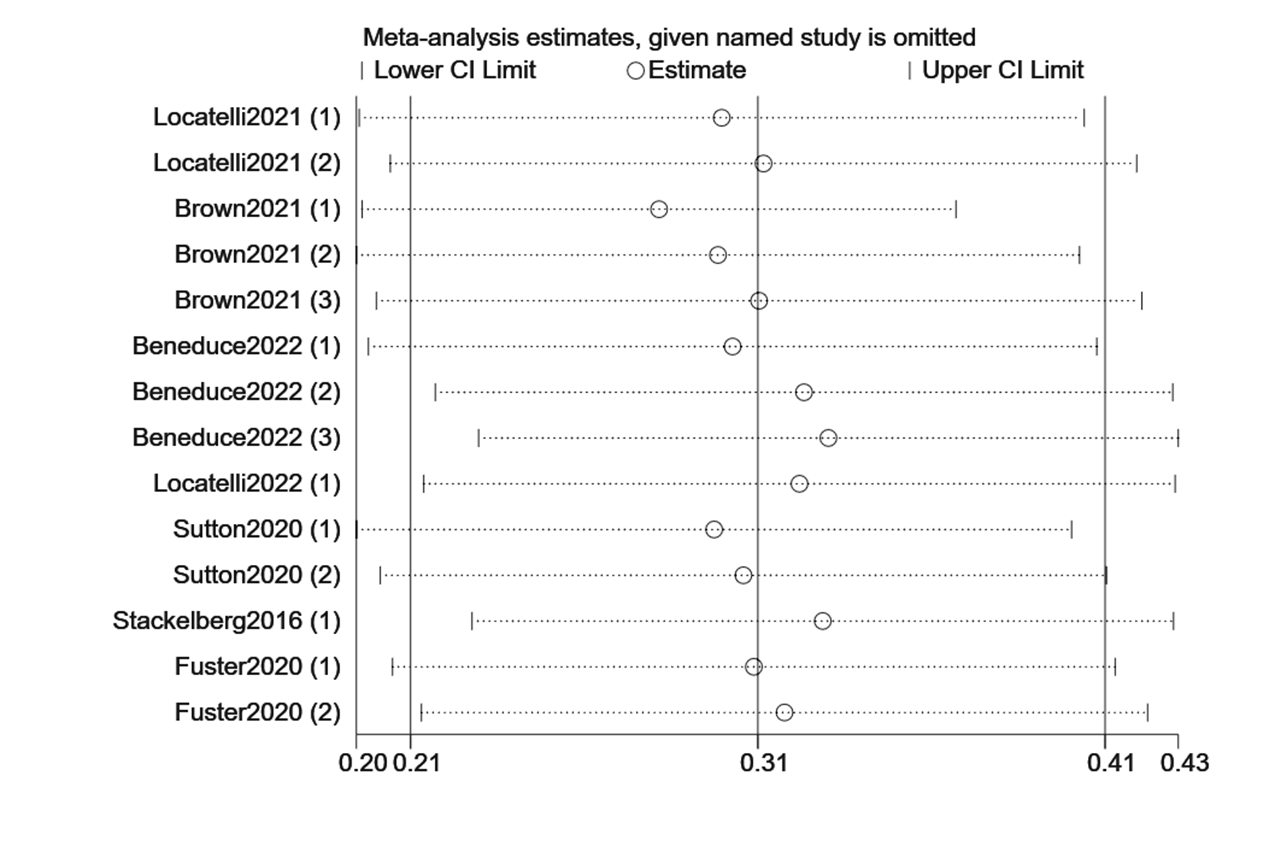


**C:** Sensitivity analysis of EFS (single-arm studies). EFS, event-free survival; 1, first year; 2, second year; 3, third year.


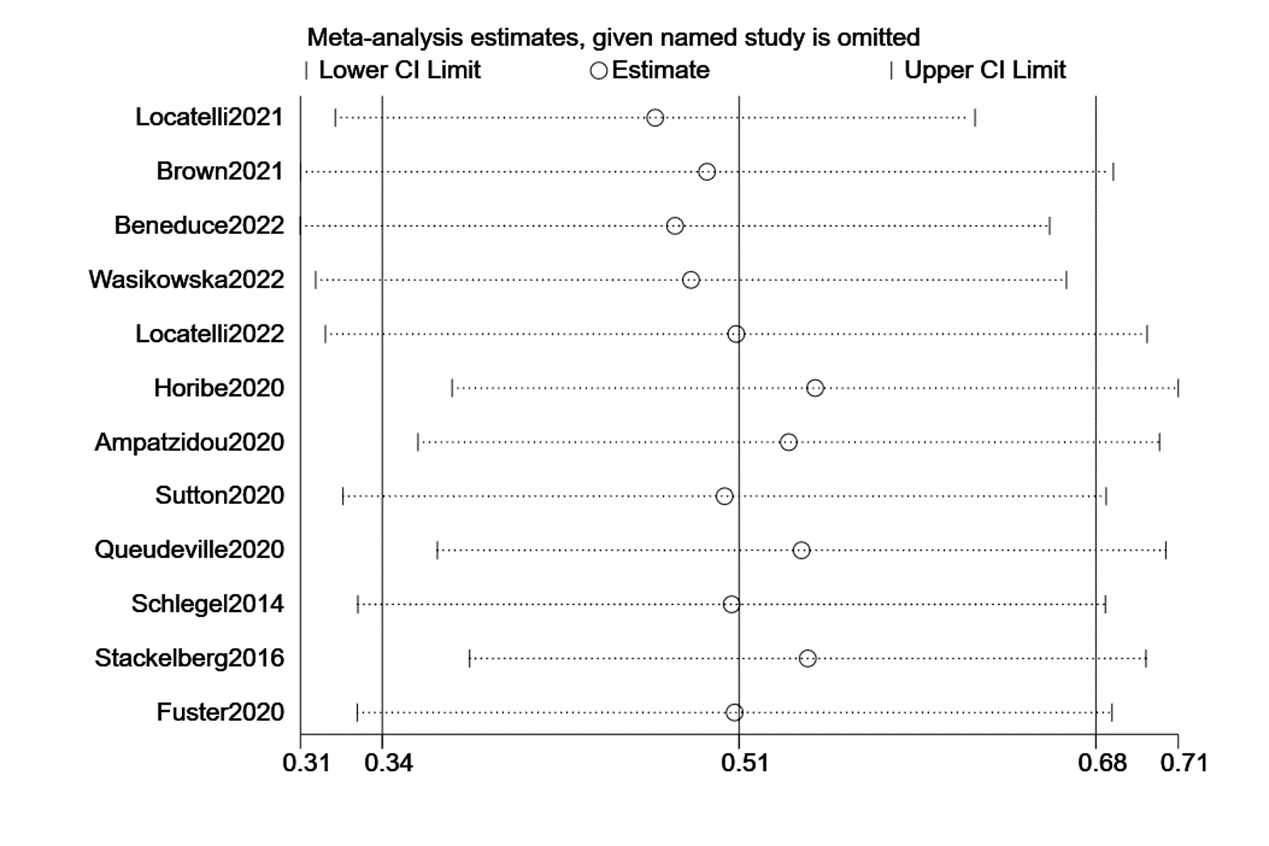


**D:** Sensitivity analysis of MRD (single-arm studies). MRD, minimal residual disease.


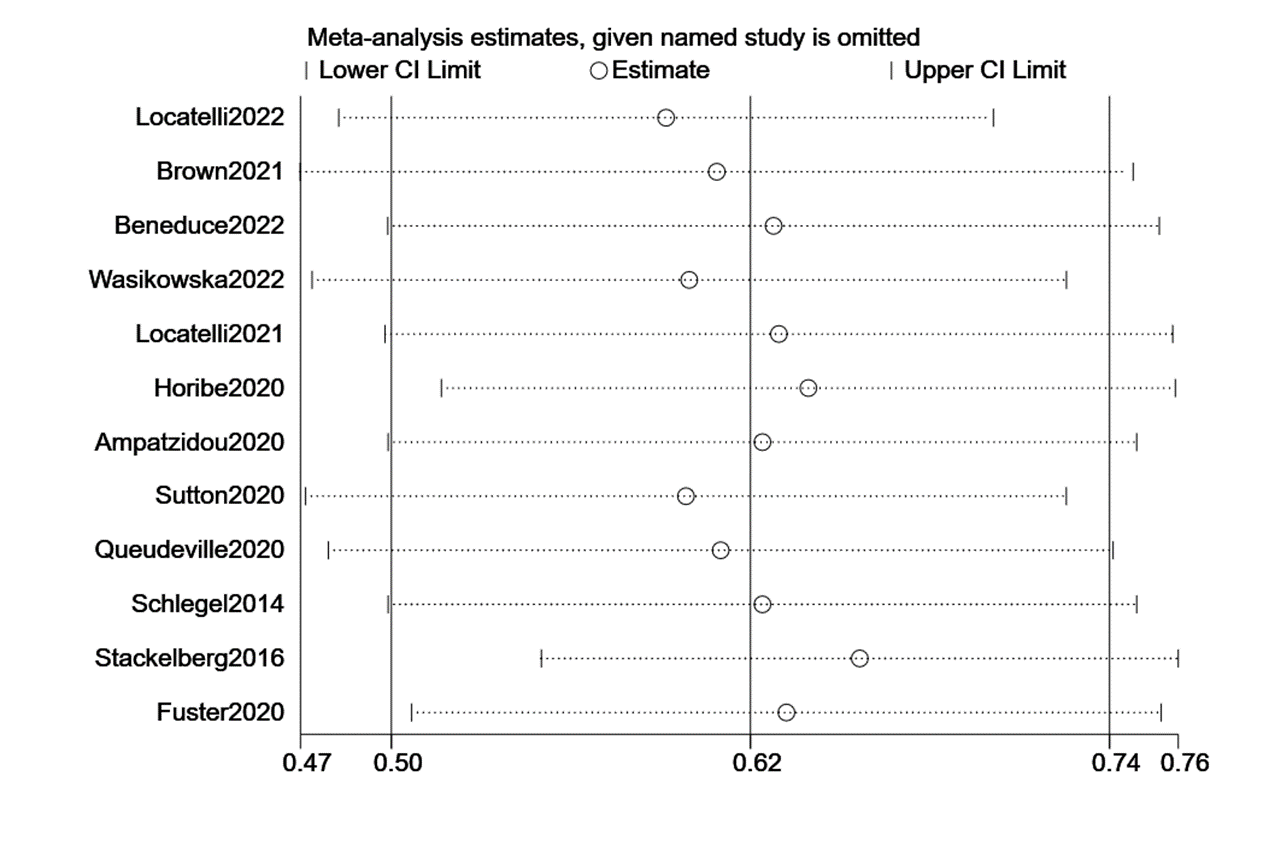


**E:** Sensitivity analysis of all0-HSCT (single-arm studies). all0-HSCT, allogeneic hematopoietic stem cell transplantation.


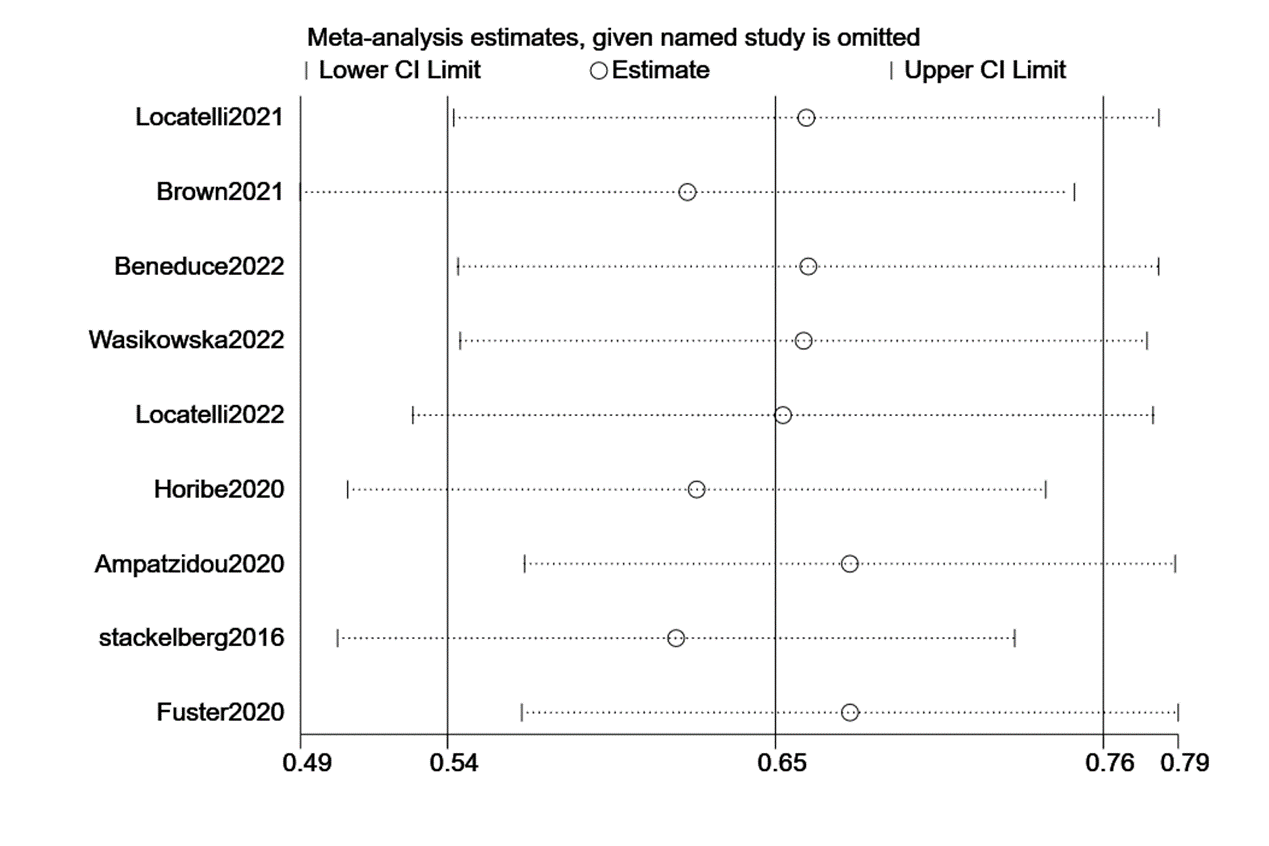


**F:** Sensitivity analysis of AE (single-arm studies). AE, adverse effects.


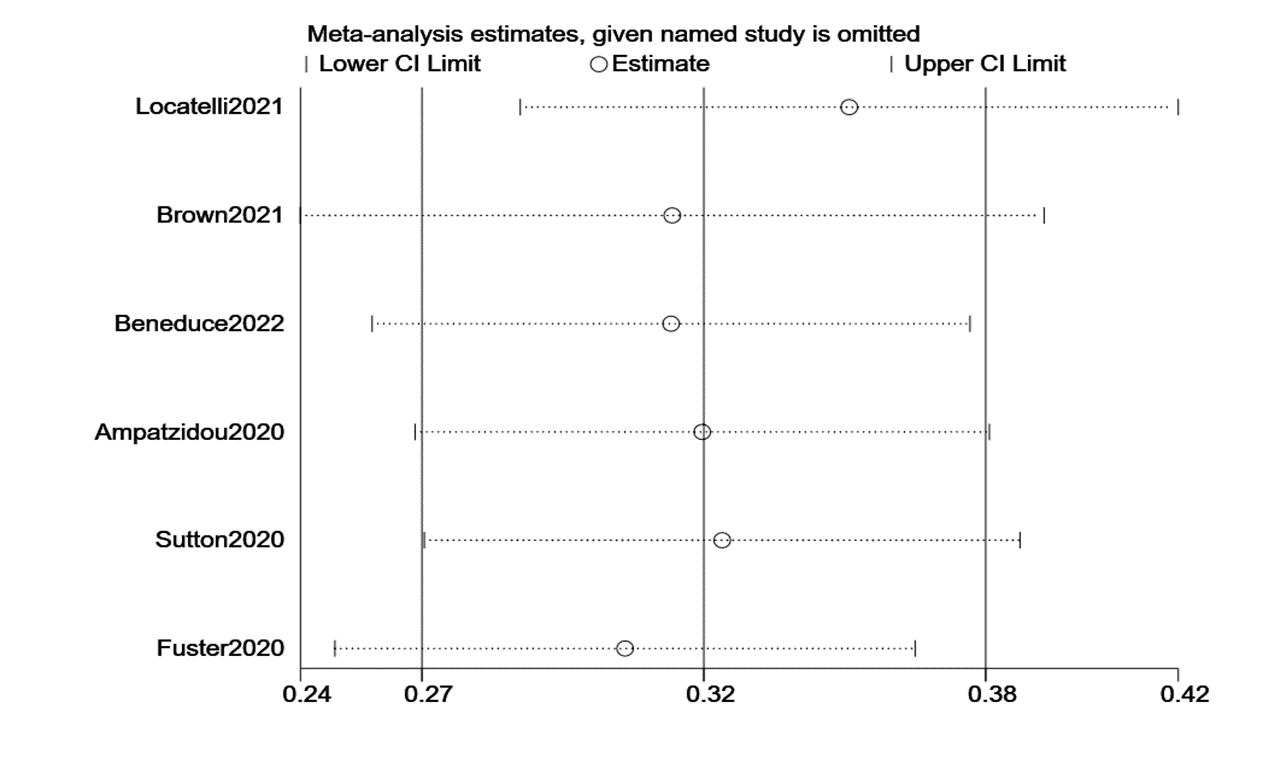


F**:** Sensitivity analysis of relapse (single-arm studies).

**Supplementary Data 6:** Forest plots. **(A)** Forest plots of tumor burden (CR); **(B)** Forest plots of allo-HSCT(RCTs); **(C)** Forest plots of allo-HSCT (single-arm studies); **(D)** Forest plots of relapse (RCTs); **(E)** Forest plots of relapse (single-arm studies). CR, complete response; allo-HSCT, allogeneic hematopoietic stem cell transplantation; RCTs, randomized clinical trials.


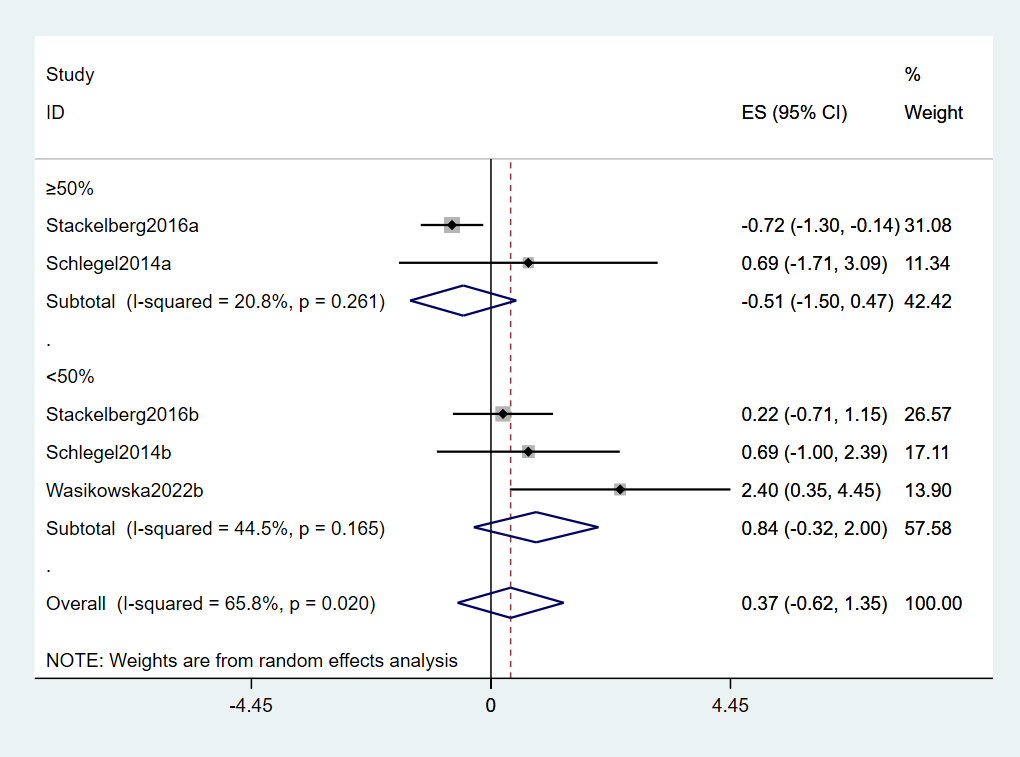


**A:** Funnel plots of tumor burden (CR). CR, complete response; 95% CI, 95% confidence interval; ES: Effect Size. The size of the rectangle at the center of the horizontal bar is proportional to the weight of the given study. The diamond at the bottom indicates the pooled ES.


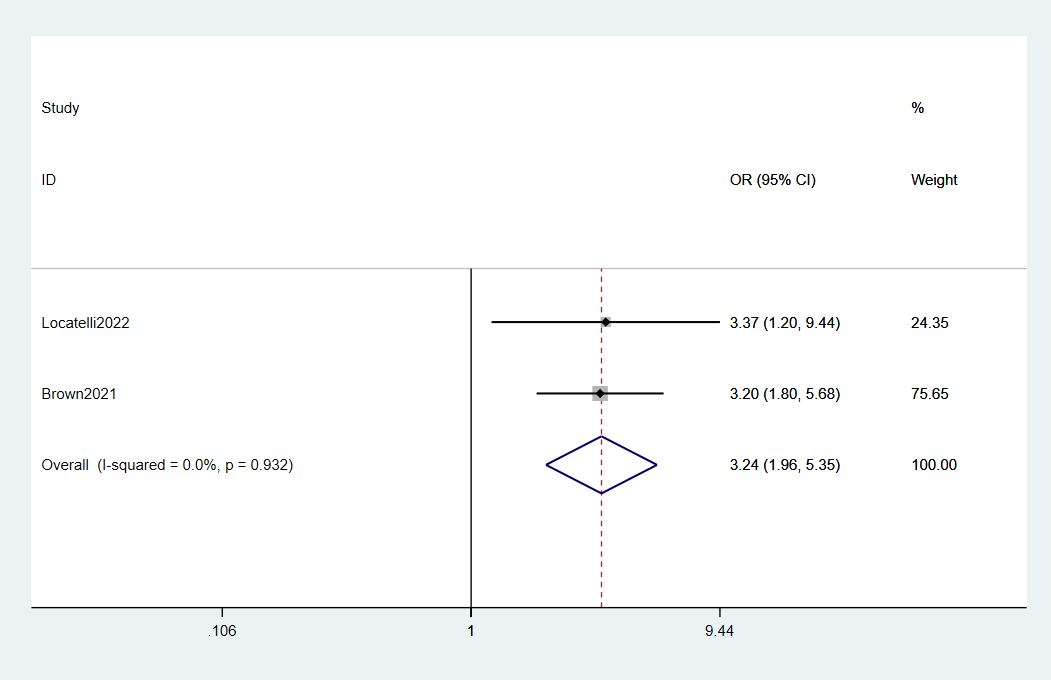


**B:** Forest plot of allo-HSCT (RCTs). all0-HSCT, allogeneic hematopoietic stem cell transplantation; RCTs, randomized clinical trials; 95% CI, 95% confidence interval; OR: odds ratio. The size of the rectangle at the center of the horizontal bar is proportional to the weight of the given study. The diamond at the bottom indicates the pooled OR.


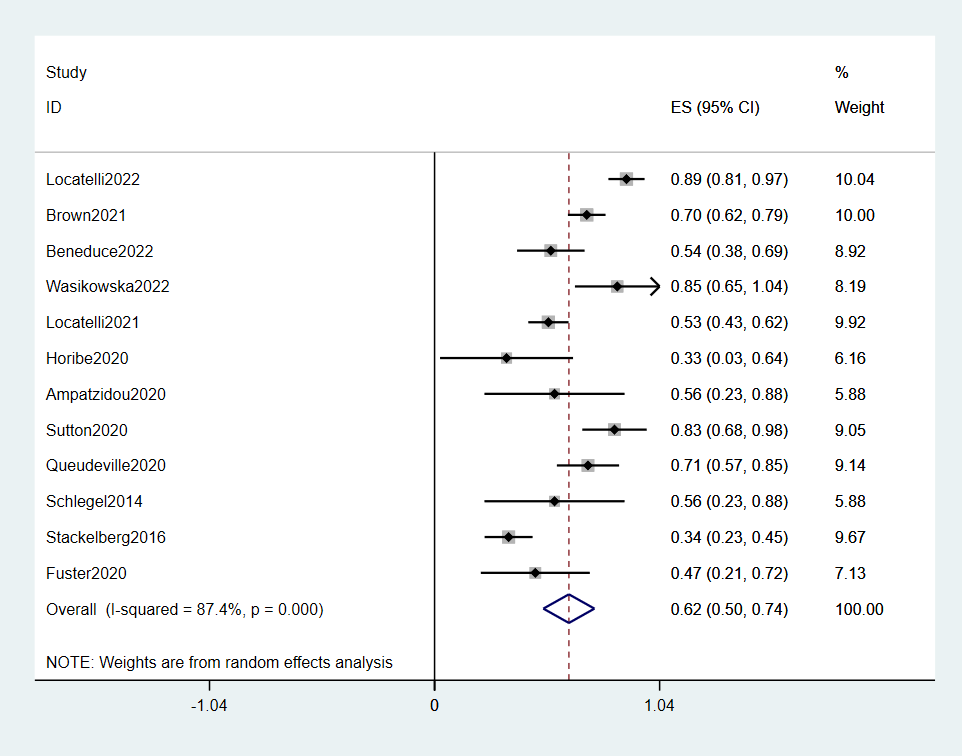


**C:** Forest plot of allo-HSCT (single-arm studies). all0-HSCT, allogeneic hematopoietic stem cell transplantation; 95% CI, 95% confidence interval; ES: Effect Size. The size of the rectangle at the center of the horizontal bar is proportional to the weight of the given study. The diamond at the bottom indicates the pooled ES.


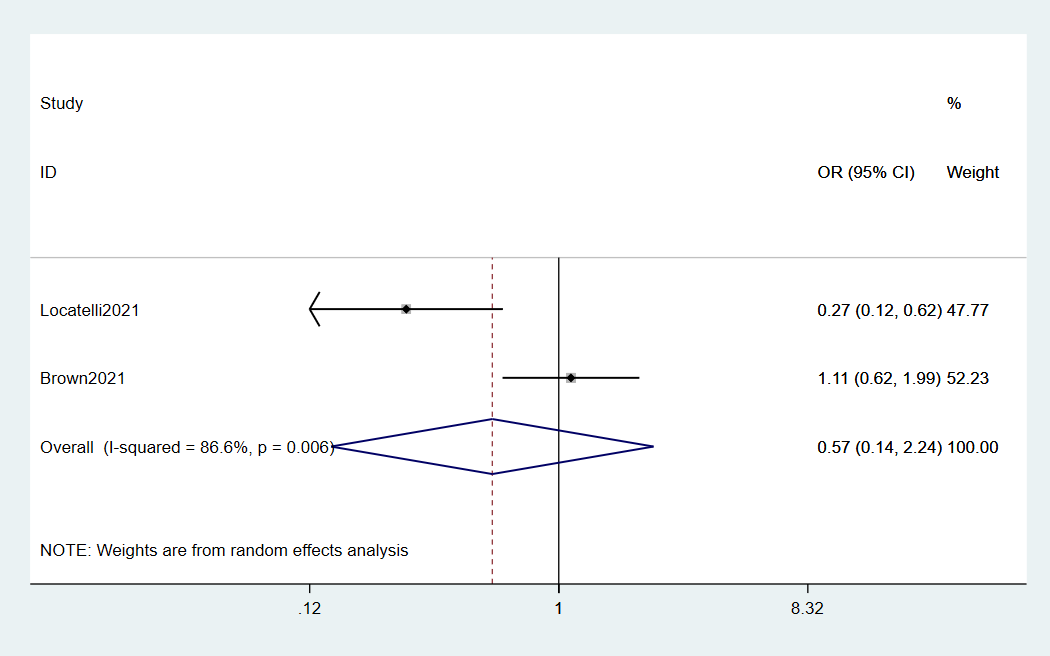


**D:** Forest plot of relapse (RCTs). AE, adverse effects；RCTs, randomized clinical trials; 95% confidence interval; OR: odds ratio. The size of the rectangle at the center of the horizontal bar is proportional to the weight of the given study. The diamond at the bottom indicates the pooled OR.


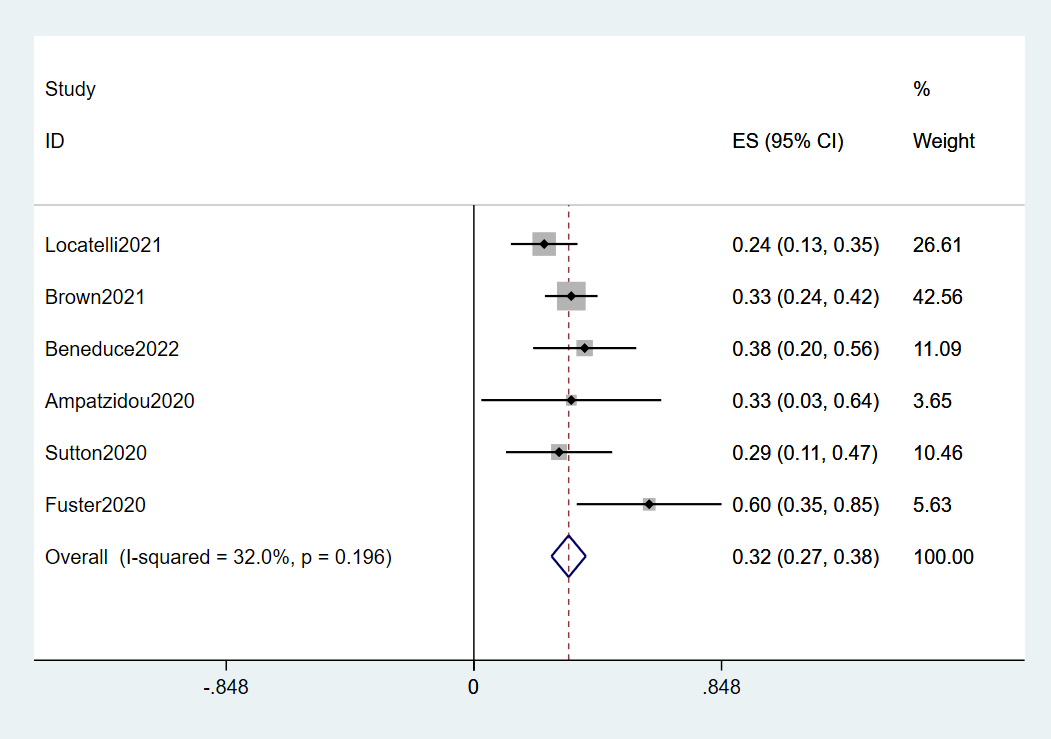


**E:** Forest plot of relapse (single-arm studies). 95% CI, 95% confidence interval; ES: Effect Size. The size of the rectangle at the center of the horizontal bar is proportional to the weight of the given study. The diamond at the bottom indicates the pooled ES.

**Supplementary Data 7:** Funnel plots of MRD bias. MRD, minimal residual disease.


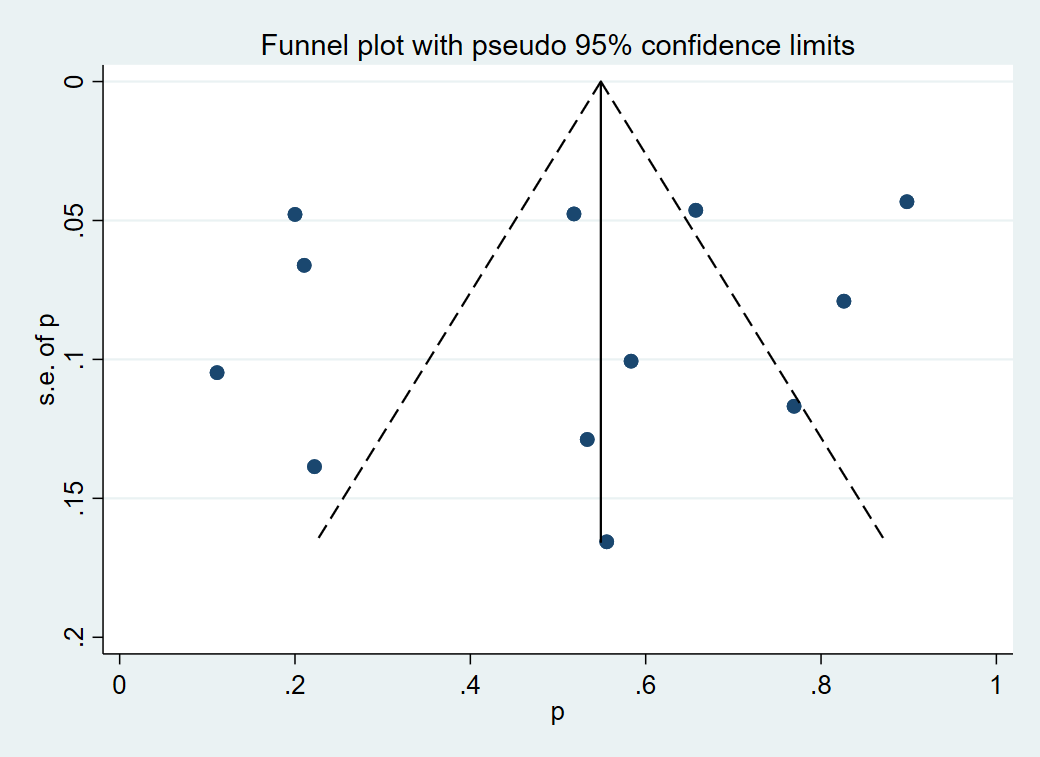


Funnel plots of MRD bias. MRD, minimal residual disease.

**Supplementary Data 8:** Egger’s test of publication bias of MRD. MRD, minimal residual disease.


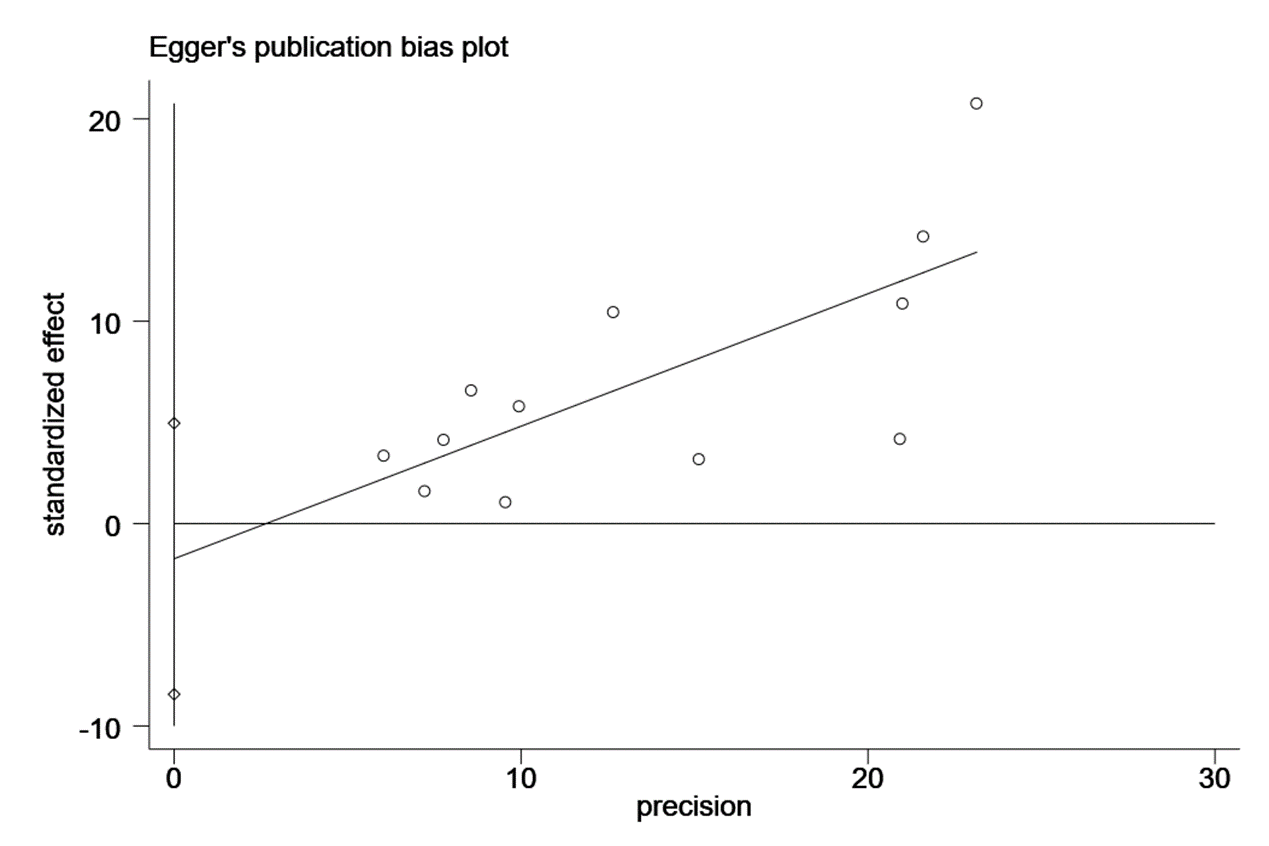


Egger’s test of publication bias of MRD. MRD, minimal residual disease.
